# Effects of riboflavin and desferrioxamine B on Fe(II) oxidation by O_2_

**DOI:** 10.1016/j.fmre.2021.09.012

**Published:** 2021-10-21

**Authors:** Peng Zhang, Philippe Van Cappellen, Kunfu Pi, Songhu Yuan

**Affiliations:** a State Key Laboratory of Biogeology and Environmental Geology, China University of Geosciences, 68 Jincheng Street, East Lake High-Tech Development Zone, Wuhan, Hubei 430078, China; b Ecohydrology Research Group, Water Institute and Department of Earth and Environmental Sciences, University of Waterloo, Waterloo, Ontario N2L 3G1, Canada; c Hubei Key Laboratory of Yangtze Catchment Environmental Aquatic Science, School of Environmental Studies, China University of Geosciences, 68 Jincheng Street, East Lake High-Tech Development Zone, Wuhan, Hubei 430078, China

**Keywords:** Riboflavin, Desferrioxamine B, Ferrous iron, Abiotic oxidation, Oxygen

## Abstract

Flavins and siderophores secreted by various plants, fungi and bacteria under iron (Fe) deficient conditions play important roles in the biogeochemical cycling of Fe in the environment. Although the mechanisms of flavin and siderophore mediated Fe(III) reduction and dissolution under anoxic conditions have been widely studied, the influence of these compounds on Fe(II) oxidation under oxic conditions is still unclear. In this study, we investigated the kinetics of aqueous Fe(II) (17.8 μM) oxidation by O_2_ at pH 5‒7 in the presence of riboflavin (oxidized (RBF) and reduced (RBFH_2_)) and desferrioxamine B (DFOB) as representative flavins and siderophores, respectively. Results showed that the addition of RBF/RBFH_2_ or DFOB markedly accelerates the oxidation of aqueous Fe(II) by O_2_. For instance, at pH 6, the rate of Fe(II) oxidation was enhanced 20‒70 times when 10 μM RBFH_2_ was added. The mechanisms responsible for the accelerated Fe(II) oxidation are related to the redox reactivity and complexation ability of RBFH_2_, RBF and DFOB. While RBFH_2_ does not readily complex Fe(II)/Fe(III), it can activate O_2_ and generate reactive oxygen species, which then rapidly oxidize Fe(II). In contrast, both RBF and DFOB do not reduce O_2_ but react with Fe(II) to form RBF/DFOB-complexed Fe(II), which in turn accelerates Fe(II) oxidation. Furthermore, the lower standard reduction potential of the Fe(II)-DFOB complex, compared to the Fe(II)-RBF complex, correlates with a higher oxidation rate constant for the Fe(II)-DFOB complex. Our study reveals an overlooked catalytic role of flavins and siderophores that may contribute to Fe(II)/Fe(III) cycling at oxic-anoxic interfaces.

## 1 Introduction

Ferrous iron (Fe(II)) is an ubiquitous Fe species in subsurface environments. Fe(II) is soluble at circumneutral pH, so it is highly bioavailable [1]. Also, due to its high reactivity, Fe(II) affects the mineral transformation [2], the biogeochemical cycling of redox-sensitive elements including carbon, nitrogen, oxygen and sulfur [1], and the natural attenuation of pollutants such as hexavalent chromium and chlorinated compounds [3]. Although the anoxic subsurface environments favor the generation and preservation of Fe(II), the redox conditions are oftentimes disturbed by O_2_ in natural and artificial processes (e.g., surface water and groundwater interaction, bank filtration, etc.) [4,5]. The presence of O_2_ results in Fe(II) oxidation to Fe(III) through abiotic and biotic pathways [1]. Because Fe(III) oxyhydroxides have high surface areas, they can adsorb trace metals and some organic compounds [3]. Overall, the oxidation of Fe(II) plays an important role in the biogeochemical cycling of redox-sensitive elements and the fate and transport of pollutants in subsurface environments.

Given the great environmental significance of Fe(II) oxidation, the mechanisms and kinetics of Fe(II) oxidation have been widely studied [6], [7], [8], [9]. In aqueous solution, the mechanism of Fe(II) oxidation by O_2_ is described as (1), (2), (3), (4) [6,8] and the rate of abiotic Fe(II) oxidation depends on the solution pH and oxygen concentration [6,7]. The pH dependence relationship is in nature ascribed to changing Fe(II) species. At low pH, free Fe^2+^ is the dominant Fe(II) species and the rate of Fe(II) oxidation is slow. As pH rises, the fraction of hydrolyzed Fe(II) species (e.g., FeOH^+^ and Fe(OH)_2_^0^) in total Fe(II) increases, and then the rate of Fe(II) oxidation also increases.

(1) Fe(II) + O_2_ → Fe(III) + ·O_2_^−^

(2) Fe(II) + ·O_2_^−^ + 2H^+^ → Fe(III) + H_2_O_2_

(3) Fe(II) + H_2_O_2_ → Fe(III) + ·OH + OH^−^

(4) Fe(II) + ·OH → Fe(III) + OH^−^

When aqueous Fe(II) is oxidized to Fe(III), the bioavailability of Fe will decrease because of the limited solubility of Fe(III) oxyhydroxides [1]. Under Fe deficient conditions, some plants (e.g., *Beta vulgaris*), fungi (e.g., *Aspergillus glaucus*) and bacteria (e.g., *Shewanella oneidensis*) secrete flavins and siderophores to elevate the concentration of aqueous Fe(II)/Fe(III) [10], [11], [12], [13]. Previous studies have reported that flavins have versatile biogeochemical redox functions [14,15]. For instance, flavins can mediate electron transfer from microbes and plant roots to Fe(III) minerals resulting in their reductive dissolution under anoxic conditions [1,16,17]. Siderophores have high affinity for Fe(III) and therefore solubilize Fe from Fe(III) minerals [11,13].

In comparison with Fe(III) reduction and dissolution under anoxic conditions, the influence of flavins and siderophores on aqueous Fe(II) oxidation under oxic conditions has received less attention. In natural systems, the soluble flavins mainly occur in reduced and oxidized forms [18], and siderophores are usually in the oxidized form. Previous studies have reported that both oxidized flavins and siderophores can react with Fe(II) forming complexed Fe(II) [19,20]. Because the reactivity of complexed Fe(II) differs from inorganic Fe(II), the presence of oxidized flavins and siderophores may affect Fe(II) oxidation. In addition, reduced flavins can activate O_2_ to generate reactive oxygen species (ROS) [14,21]. ROS is a stronger oxidant than O_2_ for Fe(II) [6,8], so the generated ROS may accelerate Fe(II) oxidation. However, reduced flavins can also reduce Fe(III) oxyhydroxides to Fe(II) [22,23], which results in Fe(II) regeneration.

The goal of this study was to reveal the influence of flavins and siderophores on the kinetics of aqueous Fe(II) oxidation by O_2_. Riboflavin (oxidized: RBF, and reduced: RBFH_2_) was chosen as the representative flavins because it is the substrate to synthesize flavin mononucleotide and flavin adenine dinucleotide [14]. The typical concentration of flavins falls in picomolar (pM) to nanomolar (nM) range in natural aquatic environments [24], and reaches up to micromolar (μM) level in microbial cultures [16] and around plant roots [17]. DFOB was a representative of hydroxamate siderophores and was also extensively studied in previous researches [11,19,20]. The concentration of hydroxamate siderophores is estimated between nM and μM level in soil solutions [25]. The effects of RBF and DFOB on aqueous Fe(II) oxidation were, respectively, assessed over the pH range of 5 to 7. A speciation calculation was conducted to reveal Fe(II) speciation in the presence of RBF and DFOB. Kinetic models were developed to represent the reactions in the inorganic Fe(II), Fe(II)-reduced RBF, Fe(II)-oxidized RBF and Fe(II)-DFOB systems.

## 2 Materials and methods

### 2.1 Chemicals

RBF, piperazine-N, N-bis (ethanesulfonic acid) sodium salt (PIPES) and 2-(N-morpholino) ethanesulfonic acid (MES) were obtained from Sigma-Aldrich. Ferrous chloride, 4-hydrate (FeCl_2_·4H_2_O) was purchased from J.T. Baker, deferoxamine mesylate from VWR International, and catalase and superoxide dismutase (SOD) from Fisher Scientific. Because RBF and DFOB were already in oxidized state, they were used as purchased. Reduced RBF (RBFH_2_) was prepared via the dithionite reduction method [22], and it was stored in an anaerobic glovebox (95% N_2_ and 5% H_2_, COY, USA) prior to use. All other chemicals were of or above analytical grade. Experimental solutions were prepared with 18.2 MΩcm Milli-Q water.

### 2.2 Batch experiments

A series of batch experiments were conducted in 125 mL serum bottles at 22 ± 1 °C. The serum bottles contained Teflon-coated magnetic stirring bars, which were used to maintain the stirring rate at 400 rpm. The bottles were wrapped by aluminum foil to exclude light and were exposed to air through several pores in the top. Prior to the experiments, 100 mL of solution containing 20 mM buffer and 10 mM NaCl was added into the bottles. The following buffers were used to control solution pH: MES for pH 5 and 6, PIPES for pH 7. MES and PIPES were chosen because they do not form complexes with Fe(II) or Fe(III) [26]. The typical experiments were initiated by simultaneously adding 17.8 μM inorganic Fe(II) (as FeCl_2_) and 10 μM RBFH_2_/RBF or DFOB into the bottles.

To determine the effect of oxidized RBF concentration on Fe(II) oxidation, RBF concentrations of 1, 2, 5 and 10 μM were, respectively, added into a 100 mL solution containing 17.8 μM inorganic Fe(II), 20 mM buffer and 10 mM NaCl. These experiments were conducted at pH 5, 6 and 7. To determine the effect of DFOB concentration on Fe(II) oxidation, DFOB concentrations of 1, 2, 5 and 10 μM were added into a 100 mL solution containing 17.8 μM inorganic Fe(II), 20 mM buffer and 10 mM NaCl. These experiments were conducted at pH 7. Control experiments were carried out without addition of RBFH_2_/RBF or DFOB but under otherwise identical conditions.

The stock solutions of RBFH_2_, RBF and DFOB were prepared every week to ensure that the concentration variation in stock solution was negligible. Solution pH varied by <0.1 unit during all the treatments. Each assay lasted 180 min. At predetermined time intervals, 180-μL sample was taken out from the bottles and was mixed rapidly with 10 μL of 10 mM ferrozine to inhibit the further oxidation of Fe(II) by O_2_. Note that ferrozine chelates all aqueous Fe(II), including RBF or DFOB complexed Fe(II) [27]. All the experiments were carried out in duplicate.

### 2.3 Quenching experiments

Previous studies have reported that ROS including superoxide radicals (·O_2_^−^), hydroxyl peroxides (H_2_O_2_) and hydroxyl radicals (·OH) are involved in Fe(II) oxidation by O_2_ [6,8,9]. To explore the roles of ·O_2_^−^ and H_2_O_2_ in Fe(II) oxidation in Fe(II)-RBF system, 100 U/L SOD and 100 mg/L catalase were separately added into the Fe(II)-RBF solution containing 17.8 μM inorganic Fe(II), 10 μM RBF, 20 mM buffer and 10 mM NaCl. SOD and catalase were used to quench ·O_2_^−^ and H_2_O_2_, respectively. These experiments were conducted at pH 6 and 7. Control experiments were conducted without RBF but under otherwise identical conditions.

### 2.4 Chemical analysis

Aqueous Fe(II) concentrations were measured by the ferrozine method at 562 nm [28] using a Flexstation-3 Multimode Reader (Molecular Devices). Our previous study has proven that the presence of RBF and Fe(III) has negligible influence on Fe(II) measurement [27]. The concentration variation of uncomplexed RBF during the reaction course was measured by the UV-vis spectrum within 300‒600 nm.

### 2.5 Kinetic modeling

Oxidation kinetics of aqueous Fe(II) under various experimental conditions were modeled numerically using the Kintecus 6.51 software [29]. The reaction networks are shown in Table 1, which are made up of three subsections: a basic section for inorganic Fe(II) oxidation (Reactions A1–A5), two individually extended sections for Fe(II) oxidation in the presence of RBFH_2_/RBF (Reactions B1–B22) and DFOB (Reactions C1–C8). More details are given in Section S1 in supporting information. In the kinetic model, multiple side reactions including the oxidation of Fe(II) catalyzed by Fe(III) oxyhydroxides, the oxidation of Fe(II), RBFH_2_, RBF and DFOB by ·OH, the complexation of Fe^2+^/Fe^3+^ by RBFH_2_/RBFH^−^, the multistep equilibrium reactions for uncomplexed DFOB, Fe^2+^-DFOB and Fe^3+^-DFOB complexes, the oxidation of Fe(II) by DFOB and the reduction of Fe(III) by DFOB are not included because these reactions were of minor importance under experimental conditions (for details, see Section S2).

**Table 1:** **Reactions and kinetic parameters for Fe(II) oxidation**.

No.	Reactions	Rate constant (or)			Source
		pH 5	pH 6	pH 7	
Reactions in inorganic Fe(II) system					
A1	Fe(II) + O_2_ → Fe(III) + ·O_2_^−^	1 × 10^−3^ M^−1^s^−1^	6 × 10^−3^ M^−1^s^−1^	3.7 × 10^−1^ M^−1^s^−1^	This study
A2	Fe(II) + ·O_2_^−^ → Fe(III) + H_2_O_2_	1 × 10^7^ M^−1^s^−1^			[9]
A3	Fe(II) + H_2_O_2_ → Fe(III) + ·OH + OH^−^	5.5 × 10^1^ M^−1^s^−1^	6.2 × 10^2^ M^−1^s^−1^	4.79 × 10^3^ M^−1^s^−1^	[9,37]
A4	Fe(III) + ·O_2_^−^ → Fe(II) + O_2_	1.5 × 10^8^ M^−1^s^−1^			[8]
A5	Fe(III) + Fe(III) (or Fe(OH)_3_) → 2Fe(OH)_3_	3 × 10^6^ M^−1^s^−1^	3 × 10^6^ M^−1^s^−1^	3.4 × 10^6^ M^−1^s^−1^	[42]
Extended reactions in Fe(II)-RBFH_2_/RBF systems					
B1	RBFH_2_ → RBFH^−^ + H^+^	1.12 × 10^−3^ s^−1^			This study
B2	RBFH^−^ + H^+^ → RBFH_2_	2 × 10^3^ M^−1^s^−1^			This study
B3	RBFH^−^ + O_2_ → RBFHOOH	2.5 × 10^2^ M^−1^s^−1^			[21]
B4	RBFHOOH → RBF + H_2_O_2_	2.6 × 10^2^ s^−1^			[21]
B5	RBFH_2_ + RBF → 2·RBFH	1 × 10^6^ M^−1^s^−1^			[21]
B6	·RBFH + ·RBFH → RBFH_2_ + RBF	5 × 10^8^ M^−1^s^−1^			[21]
B7	·RBFH + O_2_ → RBF + ·O_2_^−^	1 × 10^4^ M^−1^s^−1^			[21]
B8	·RBFH + ·O_2_^−^ → RBF + H_2_O_2_	1 × 10^8^ M^−1^s^−1^			[21]
B9	RBFH_2_ (or RBFH^−^) + ·O_2_^−^ →·RBFH + H_2_O_2_	1.8 × 10^5^ M^−1^s^−1^			[12]
B10	RBFH_2_ (or RBFH^−^) + Fe(III) (or Fe(OH)_3_) → ·RBFH + Fe(II)	4.4 × 10^2^ M^−1^s^−1^			This study
B11	Fe(II) + RBF → Fe^2+^-RBF^−^ + H^+^	8.0 × 10^4^ M^−1^s^−1^			[27]
B12	Fe^2+^-RBF^−^ + H^+^ → Fe(II) + RBF	1 × 10^9^ M^−1^s^−1^			[27]
B13	Fe(III) + RBF → Fe^3+^-RBF^−^ + H^+^	6.4 × 10^5^ M^−1^s^−1^			[27]
B14	Fe^3+^-RBF^−^ + H^+^ → Fe(III) + RBF	2 × 10^2^ M^−1^s^−1^			[27]
B15	Fe(II) + RBF → Fe(III) + ·RBFH	2 × 10^−6^ M^−1^s^−1^			[27]
B16	Fe(II) + ·RBFH→ Fe(III) + RBFH_2_	3 × 10^−6^ M^−1^s^−1^			[27]
B17	Fe^2+^-RBF^−^ → Fe(III) + ·RBFH	5 × 10^−4^ s^−1^			[27]
B18	Fe^2+^-RBF^−^ + O_2_ → Fe(III) + ·RBFOO^−^	4 × 10^2^ M^−1^s^−1^	3.3 × 10^2^ M^−1^s^−1^	2.1 × 10^2^ M^−1^s^−1^	This study
B19	Fe^2+^-RBF^−^ + ·O_2_^−^ → Fe^3+^-RBF^−^ + H_2_O_2_	1 × 10^7^ M^−1^s^−1^			[9]
B20	Fe^2+^-RBF^−^ + H_2_O_2_ → Fe^3+^-RBF^−^ + ·OH + OH^−^	5.5 × 10^1^ M^−1^s^−1^	6.2 × 10^2^ M^−1^s^−1^	4.79 × 10^3^ M^−1^s^−1^	[9,37]
B21	Fe^2+^-RBF^−^ (or Fe(II)) + ·RBFOO^−^ → Fe^3+^-RBF^−^ (or Fe(III)) + RBFHOOH	1 × 10^4^ M^−1^s^−1^			This study
B22	Fe^3+^-RBF^−^ + ·O_2_^−^ → Fe^2+^-RBF^−^ + O_2_	1.5 × 10^8^ M^−1^s^−1^			[8]
Extended reactions in Fe(II)-DFOB system					
C1	Fe(II) + DFOB → Fe^2+^-DFOB	3.2 × 10^4^ M^−1^s^−1^			This study
C2	Fe^2+^-DFOB → Fe(II) + DFOB	1.5 s^−1^			This study
C3	Fe(III) + DFOB → Fe^3+^-DFOB	1.96 × 10^6^ M^−1^s^−1^			[39]
C4	Fe^3+^-DFOB → Fe(III) + DFOB	1.5 × 10^−6^ s^−1^			[39]
C5	Fe^2+^-DFOB + O_2_ → Fe^3+^-DFOB + ·O_2_^−^	5 × 10^2^ M^−1^s^−1^	3 × 10^4^ M^−1^s^−1^	5 × 10^4^ M^−1^s^−1^	This study
C6	Fe^2+^-DFOB + ·O_2_^−^ → Fe^3+^-DFOB + H_2_O_2_	1 × 10^7^ M^−1^s^−1^			[9]
C7	Fe^2+^-DFOB + H_2_O_2_ → Fe^3+^-DFOB + ·OH + OH^−^	1 × 10^4^ M^−1^s^−1^	2 × 10^5^ M^−1^s^−1^	3 × 10^5^ M^−1^s^−1^	This study
C8	Fe^3+^-DFOB + ·O_2_^−^ → Fe^2+^-DFOB + O_2_	1.5 × 10^8^ M^−1^s^−1^			[8]

Most of the rate constants were cited from literature. To achieve the optimal fitting, some rate constants were adjusted or fitted (for details, see Section S1). For instance, the rate constant for aqueous Fe(III) hydrolysis has been reported to be 3.2 × 10^5^ M^−1^s^−1^ at pH 6 [42]. The value was adjusted to 3 × 10^6^ M^−1^s^−1^ to account for the relatively high Fe(III) concentration in this study (Section S1). In addition, to reduce the number of fitting parameters, we assumed that the rate constants for the reactions between complexed Fe(II) and ·O_2_^−^/H_2_O_2_ were equal to those for inorganic (i.e., uncomplexed) Fe(II) (Section S1). Due to minor variation of solution pH (<0.1 unit) during all experiments, constant pH values were imposed in the calculations. Data obtained at the three different pH values (5, 6, and 7) were fitted separately. The relative importance of each pathway on Fe(II) oxidation was assessed by comparing the normalized sensitivity coefficients (NSCs) that were calculated following previously published methods [9,30]. The NSCs allow one to identify which rate constants in a reaction network require accurate values, that is, which are the most sensitive rate constants in the network [29]. For a given species and rate constant, the NSC is calculated as the partial derivative of the normalized concentration of the species with respect to the normalized rate constant [29]. For Fe(II) oxidation, positive NSCs indicate reactions that generate Fe(II), whilst negative NSCs indicate reactions consume Fe(II) [29,30].

### 2.6 Speciation calculation

Speciation calculations for Fe(II) under the different experimental conditions were carried out with Visual MINTEQ 3.1 [31]. We imposed the values for the dissociation constants of DFOB and RBF and the complexation constants of Fe^2+^/Fe^3+^ by RBF and DFOB listed in Tables S1 and S2. The initial concentrations of Fe^2+^, RBF and DFOB were set according to the experimental conditions. Because of the low Fe^2+^ concentrations in this study, the formation of Fe(II)-containing minerals was assumed to be negligible and not considered in the speciation model.

## 3 Results

### 3.1 Kinetics of inorganic Fe(II) oxidation at pH 5‒7

**Fig. 1:**
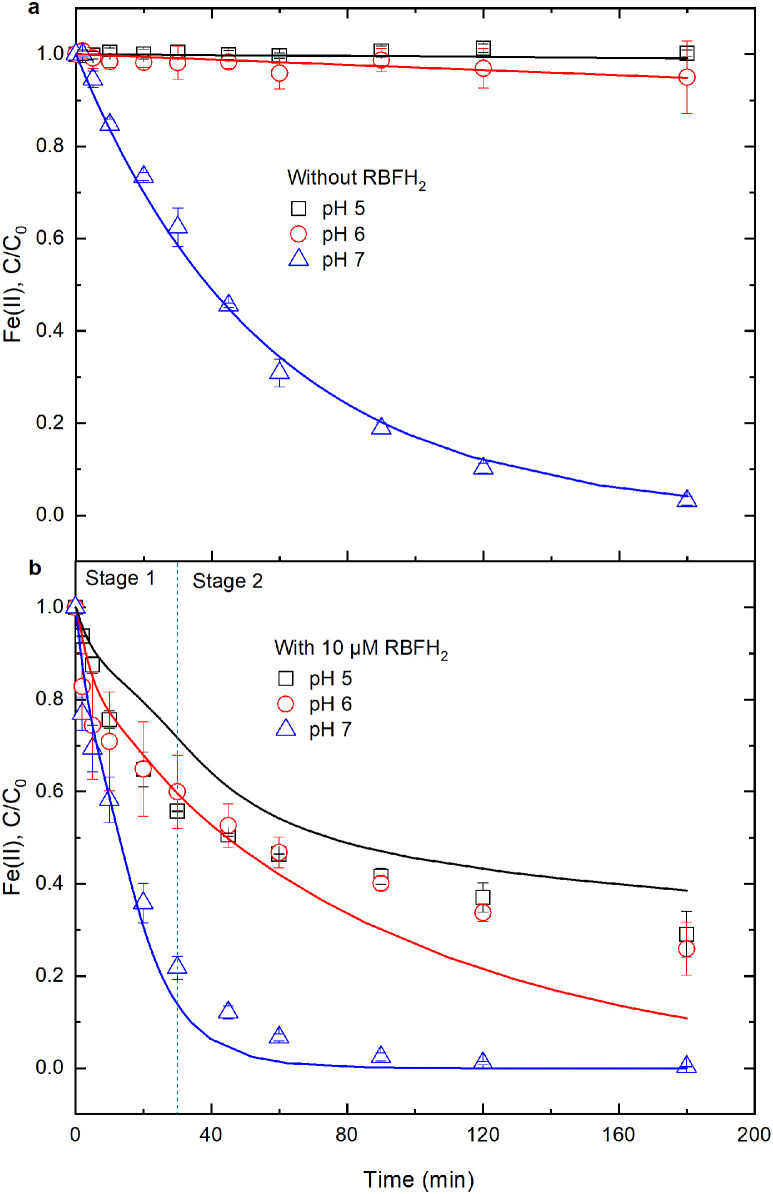
**Effect of solution pH on Fe(II) oxidation by O_2_ in Fe(II) and Fe(II)-RBFH_2_ system.** Initial conditions: RBFH_2_ concentration and solution pH specified in panels (a,b), 17.8 μM Fe(II), 10 mM NaCl and 20 mM buffer under oxic conditions. Points are the average values from duplicate experiments; lines are the modeled curves.

Prior to assessing the influence of RBF, RBFH_2_ and DFOB on Fe(II) oxidation, the inorganic Fe(II) oxidation kinetics were measured at pH 5‒7 under oxic conditions. Within 180 min, the concentrations of inorganic Fe(II) (17.8 μM initially) varied by less than 1% at pH 5, by 5% at pH 6 and by 97% at pH 7 (Fig. 1a). According to the linear regression analysis (Fig. S1), the apparent rate constants for inorganic Fe(II) oxidation at pH 6 and 7 were derived to be 3.0 × 10^−4^ and 1.9 × 10^−2^ min^−1^, respectively (Table S3). Our rate constants are in agreement with previously reported values (Fig. S2). However, the oxidation of Fe(II) at pH 5 was too slow to yield a reliable rate constant by linear fitting (Fig. S1). Herein, we used a previously reported value of 1.6 × 10^−5^ min^−1^ [6] as the apparent rate constant of Fe(II) oxidation at pH 5.

### 3.2 Effect of RBFH_2_ on inorganic Fe(II) oxidation

When 10 μM RBFH_2_ was added into above 17.8 μM Fe(II) solution, Fe(II) concentrations dropped by 71%, 74% and 100% within 180 min for pH 5, 6 and 7, respectively (Fig. 1b), which were much higher than those in the absence of RBFH_2_. So, the presence of RBFH_2_ accelerated Fe(II) oxidation by O_2_. In Fe(II)-RBFH_2_ system, Fe(II) oxidation could be divided into two stages, i.e., a quick decrease at the initial stage (Stage 1, 0‒30 min) and a slow decrease at the last stage (Stage 2, 30‒180 min) (Fig. 1b), and it followed the pseudo first-order kinetics in each stage (Fig. S1). At stage 1, the rate constants of Fe(II) oxidation were estimated to be 2.1 × 10^−2^ min^−1^ at pH 5, 2.1 × 10^−2^ min^−1^ at pH 6 and 5.2 × 10^−2^ min^−1^ at pH 7 (Table S4). At stage 2, the rate constants decreased to 4 × 10^−3^ min^−1^, 6 × 10^−3^ min^−1^ and 2.9 × 10^−2^ min^−1^ for pH 5, 6 and 7, respectively (Table S4). In comparison with inorganic Fe(II) system, Fe(II) oxidation rates were increased by 250‒1312 times at pH 5, by 20‒70 times at pH 6 and by 1.5‒2.7 times at pH 7 in Fe(II)-RBFH_2_ system.

### 3.3 Effect of RBF on inorganic Fe(II) oxidation

**Fig. 2:**
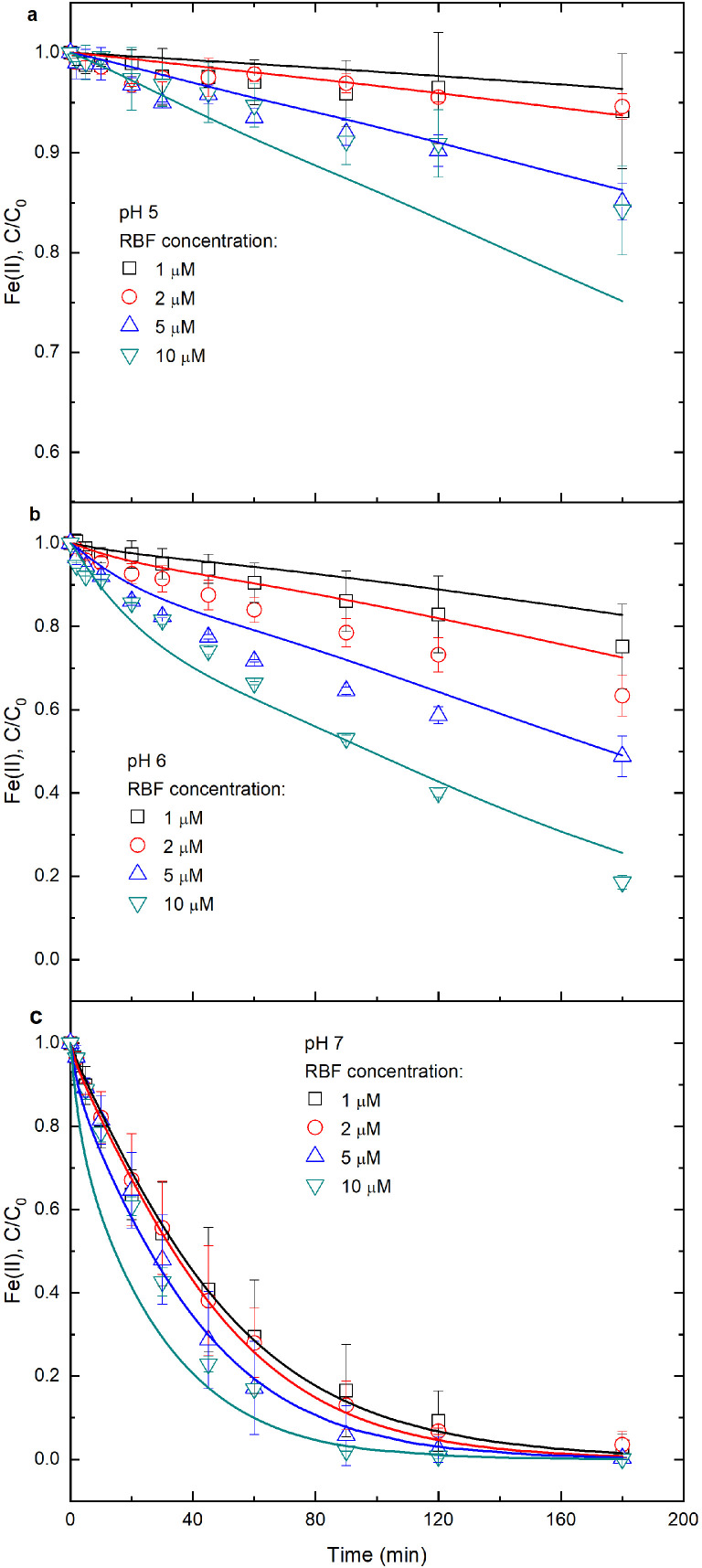
**Effect of initial RBF concentrations on Fe(II) oxidation by O_2_ at different solution pH.** Initial conditions: variable RBF concentrations and solution pH specified in panels (a‒c), 17.8 μM Fe(II), 10 mM NaCl and 20 mM buffer under oxic conditions. Points are the average values from duplicate experiments; lines are the modeled curves.

To explore the influence of RBF on Fe(II) oxidation, 10 μM RBF instead of RBFH_2_ was added into Fe(II) solution. Results show that the percentages of Fe(II) oxidation in Fe(II)-RBF system reached 16%, 81% and 100% within 180 min for pH 5, 6 and 7, respectively (Fig. 2), which were higher than those in inorganic Fe(II) system. In Fe(II)-RBF system, Fe(II) oxidation also followed the pseudo first-order kinetics (Fig. S3), giving the rate constants of 9.3 × 10^−4^, 8.4 × 10^−3^ and 3.8 × 10^−2^ min^−1^ for pH 5, 6 and 7, respectively (Table S3). So, the Fe(II) oxidation rates were enhanced by 58 times at pH 5, by 28 times at pH 6 and by 2 times at pH 7 after addition of RBF.

When RBF concentrations increased from 1 to 10 μM, the rate constants for Fe(II) oxidation increased from 3.8 × 10^−4^ to 9.3 × 10^−4^ min^−1^ at pH 5, from 1.6 × 10^−3^ to 8.4 × 10^−3^ min^−1^ at pH 6, from 2.1 × 10^−2^ to 3.8 × 10^−2^ min^−1^ at pH 7 (Table S3). In addition, at a given pH, the rate constant of Fe(II) oxidation correlated linearly with the RBF concentration (Fig. S4), in turn, suggesting that the formation of the Fe^2+^-RBF^−^ complex accelerated Fe(II) oxidation. However, the relative increase in the rate constant with increasing RBF concentration (i.e., the slopes on Fig. S4) was weakest at pH 5, highest at pH 6 and intermediate at pH7. In particular, the reduced acceleration effect of RBF at pH 7, compared to pH 6, may seem in contradiction with the much larger fractions of Fe^2+^-RBF^−^ complex at pH 7 (Fig. S5). This apparent contradiction can be explained by the pH-dependent hydrolysis of Fe(II). The latter is responsible for the large increase in the rate constant of Fe(II) oxidation between pH 6 and 7 that was observed in the absence of RBF (Table S3, Fig. S4). In other words, between pH 6 and 7, the acceleration effect due to Fe(II) hydrolysis outcompeted that of Fe^2+^-RBF^−^ complex formation. The relative effect of RBF on Fe(II) oxidation was therefore most pronounced between pH 5 and 6.

### 3.4 Roles of ·O_2_^−^ and H_2_O_2_ in Fe(II) oxidation in Fe(II)-RBF system

To evaluate whether ·O_2_^−^ and H_2_O_2_ were involved in Fe(II) oxidation in Fe(II)-RBF system, 100 U/L SOD and 100 mg/L catalase were separately added into 17.8 μM Fe(II) and 10 μM RBF solution. Results show that both SOD and catalase could observably inhibit Fe(II) oxidation (Fig. 4), which suggests that ·O_2_^−^ and H_2_O_2_ may be important oxidants for Fe(II) oxidation in Fe(II)-RBF system. In the presence of SOD, the rate constants of Fe(II) oxidation were estimated to be 3.7 × 10^−3^ min^−1^ at pH 6 and 1.9 × 10^−2^ min^−1^ at pH 7; in the presence of catalase, the rate constants of Fe(II) oxidation were 1.1 × 10^−3^ min^−1^ at pH 6 and 3.7 × 10^−3^ min^−1^ at pH 7 (Fig. S6). Based on Eq. 5, the quenching efficiencies (*QE*) of SOD on Fe(II) oxidation were estimated to be 56% at pH 6 and 50% at pH 7; while the quenching efficiencies of catalase were estimated to be 87% at pH 6 and 90% at pH 7. Control experiments show that the quenching efficiencies of SOD and catalase on inorganic Fe(II) oxidation at pH 7 were estimated to be 79% and 91%, respectively (Section S4). Hence, the quenching efficiency of SOD on Fe(II) oxidation in the Fe(II)-RBF system was lower than that in the inorganic Fe(II) system, while the quenching efficiencies of catalase were close:

(5) $$QE=\left(1-\frac{k_{i}}{k_{app}}\right)\times 100\%$$

where *k*_i_ and *k*_app_ were the rate constants for Fe(II) oxidation with and without addition of the quenchers, respectively.

### 3.5 Effect of DFOB on inorganic Fe(II) oxidation

When 10 μM DFOB was added into 17.8 μM Fe(II) solution under oxic conditions, a rapid decrease in Fe(II) was observed and the kinetics of Fe(II) oxidation could be divided into two stages, i.e., fast followed by slow (Fig. 4a). At pH 5, the Fe(II) concentration decreased by 9.1 μM (51%) within the initial 30 min, while varied negligibly in later 150 min. At pH 6 and 7, Fe(II) concentration decreased by 10.5 μM (59%) and by 11.7 μM (66%) within the initial 2 min, respectively. The net decrease of Fe(II) concentration at the initial stage was 9.1‒11.7 μM, which was close to DFOB dosage (10 μM). The curves for ln(*C*/*C*_0_) values versus reaction time were nonlinear (Fig. S8), and therefore Fe(II) oxidation in Fe(II)-DFOB system cannot be described by the pseudo first-order kinetic model, which was different from the Fe(II)-RBF/RBFH_2_ system. This difference may be attributed to the different complexation abilities of DFOB and RBF.

The effect of initial DFOB concentration on Fe(II) oxidation at pH 7 and under oxic conditions is illustrated in Fig. 4b. Fe(II) oxidation rates increased with increasing DFOB concentrations. When the initial DFOB concentration increased from 1 to 10 μM, oxidized Fe(II) within initial 2 min linearly increased from 3.2 μM (18%) to 11.7 μM (66%) (Fig. S8).

### 3.6 Results of kinetic model

The numerical modeling results for aqueous Fe(II) concentration variation versus time are illustrated in Figs. 1, 2 and 4. The model-predicted Fe(II) time trajectories are in general agreement with the observed trends. Hence, the reactions in Table 1 can be used to represent the most important reactions for aqueous Fe(II) oxidation by O_2_ in inorganic Fe(II), Fe(II)-RBFH_2_, Fe(II)-RBF and Fe(II)-DFOB systems. Also, the assumptions that were made in this study are reasonable and have marginally influenced the modeling results. However, in the Fe(II)-RBF system at pH 5, the modeled Fe(II) oxidation trajectories were below the measured concentrations. Possibly, under acidic conditions the kinetic model overestimates the relative importance of Fe(II) oxidation by RBF.

## 4 Discussion

### 4.1 Mechanism of accelerated Fe(II) oxidation by RBFH_2_

When RBFH_2_ was added into the inorganic Fe(II) solution under oxic conditions, RBFH_2_ either reacted with O_2_ generating H_2_O_2_ (reactions B1‒B9 in Table 1) or reduced Fe(III) oxyhydroxides to Fe(II) (reaction B10 in Table 1) [14,[21], [22], [23]]. However, at the initial stage, the concentration of generated Fe(III) is low, so RBFH_2_ may be oxidized predominantly by O_2_. According to the kinetic model, in the sole RBFH_2_ system, 95% of 10 μM RBFH_2_ was oxidized to RBF within 30 min at pH 5‒7 (i.e., most of RBFH_2_ could be oxidized by O_2_ at stage 1), and the steady concentration of generated H_2_O_2_ reached up to 10 μM (Fig. S9). H_2_O_2_ can oxidize rapidly Fe(II) to Fe(III) [9], so the generated H_2_O_2_ may accelerate Fe(II) oxidation. These results are in line with the experimental observation that Fe(II) was rapidly oxidized at stage 1 (0‒30 min) in Fe(II)-RBFH_2_ system (Fig. 1b). When H_2_O_2_ is exhausted at stage 1, the generated RBF may predominantly accelerate Fe(II) oxidation at stage 2.

To assess the relative importance of each reaction on Fe(II) oxidation, the matrices of NSCs at 1, 15 and 150 min were computed. The reaction times of 1 and 15 min were used to represent the initial stage, and the reaction time of 150 min represented the last stage. The results for the Fe(II)-RBFH_2_ system are shown in Fig. 5.

At times 1, 15 and 150 min, reactions A2‒A3 yielded the negative NSC values while reactions A1 and B15‒B17 were close to zero. Hence, at pH 6, inorganic Fe(II) was mainly oxidized by ·O_2_^−^ and H_2_O_2_, instead of O_2_, RBF and ·RBFH. The autodecomposition of the Fe^2+^-RBF^−^ complex had negligible contribution to Fe(II) oxidation. In the presence of RBFH_2_, ·O_2_^−^ and H_2_O_2_ were mainly produced from the oxidation of RBFH^−^ and ·RBFH by O_2_ (Table 1), so it is expected that the reactions for RBFH_2_ dissociation and the oxidation of ·RBFH by O_2_ (reactions B1 and B7) generated large negative NSC values, while the combination of two ·RBFH (reaction B6) generated a positive NSC value. For the reduction of Fe^3+^/Fe(III) oxyhydroxides by RBFH_2_/RBFH^−^ (reaction B10), it was a producer of Fe(II) and therefore generated a positive NSC value. The NSC value for reaction B10 was small at time 1 min, but was large at 15 and 150 min (Fig. 5). These results support the speculation that the relative importance of RBFH_2_/RBFH^−^ oxidation by Fe(III) increased with increasing reaction time.

At time 150 min, the oxidation of Fe^2+^-RBF^−^ complex by O_2_ (reaction B18) appeared to be the most important reaction on Fe(II) oxidation given the largest negative NSC value. Besides, the NSC value for the complexation of Fe^2+^ by RBF (reaction B11) was close to reaction B18. Hence, the oxidation of Fe^2+^-RBF^−^ complex may be the predominant pathway for Fe(II) oxidation at the last stage. The specific mechanism for Fe^2+^-RBF^−^ complex oxidation will be discussed later.

Hence, the mechanism of accelerated Fe(II) oxidation by RBFH_2_ is ascribed to the generation of ·O_2_^−^ and H_2_O_2_ through RBFH_2_/RBFH^−^ oxidation by O_2_ at the initial stage and the formation and oxidation of the Fe^2+^-RBF^−^ complex in the last stage. Fig. 1 also shows that the acceleration of Fe(II) oxidation by RBFH_2_ was dependent on the solution pH. This pH-dependence can be explained by the following reasons. First, pH affects RBFH_2_ dissociation (reaction B1 in Table 1), and then influences the generation of H_2_O_2_ from RBFH^−^ oxidation by O_2_ (reactions B3‒B4 in Table 1). Second, both the oxidation of inorganic Fe(II) by H_2_O_2_ (reaction A3 in Table 1) and the complexation of Fe^2+^ by RBF (reaction B11 in Table 1) increased with increasing pH.

### 4.2 Mechanism of accelerated Fe(II) oxidation by RBF

Previous studies have reported that RBF could function as both electron shuttle and Fe(III)-complexing ligand to mediate the reduction of Fe(III) minerals by Fe-reducing microorganisms [1,16,18]. In analogy to microbial Fe(III) reduction, we assume that RBF could also contribute to Fe(II) oxidation by O_2_ through electron shuttle and ligand effect.

#### 4.2.1 Accelerated Fe(II) oxidation through electron shuttle pathway

To achieve the electron shuttling effect between Fe(II) and O_2_, the following fundamental processes may be involved: Fe(II) is oxidized by RBF to generate Fe(III) and the resultant RBFH_2_ is re-oxidized to RBF. Our previous study showed that inorganic Fe(II) is oxidized in the Fe(II)-RBF system under anoxic conditions through the oxidation of Fe(II) by RBF and ·RBFH (reactions B15‒B16 in Table 1) and the autodecomposition of the Fe^2+^-RBF^−^ complex (reaction B17 in Table 1) [27]. The generated ·RBFH and RBFH_2_ will react with O_2_ to generate ·O_2_^−^ and H_2_O_2_ (reactions B1‒B9 in Table 1), respectively, which further contribute to Fe(II) oxidation. Thus, the electron shuttling effect may accelerate Fe(II) oxidation.

#### 4.2.2 Accelerated Fe(II) oxidation through altering Fe(II)/Fe(III) speciation

According to the kinetic model, the rate constant for Fe^2+^-RBF^−^ complex oxidation by O_2_ (reaction B14) is derived to be 2.1 × 10^2^–4 × 10^2^ M^−1^s^−1^ at pH 5‒7 (Table 1), which is several orders of magnitude higher than those for inorganic Fe(II) (reaction A1). Hence, the formation and oxidation of Fe^2+^-RBF^−^ complex may accelerate Fe(II) oxidation in Fe(II)-RBF system. Previous studies proposed that the rate constant for different Fe(II) oxidation by O_2_ depends almost linearly on its standard reduction potential (E^0^) of Fe(III)/Fe(II) couples, i.e., linear free energy relationship (LFER) [32,33]. Based on the LFER approach, we compared the rate constant for Fe^2+^-RBF^−^ complex oxidation by O_2_ with those for the inorganic and organically complexed Fe(II) species that were reported in the literature. As shown in Fig. 6, the experimental rate constant for Fe^2+^-RBF^−^ complex oxidation by O_2_ was close to the upper limit for 95% confidence intervals of linear fitting, which suggests that Fe^2+^-RBF^−^ complex oxidation by O_2_ also followed the LFER approach. Hence, the high reactivity of Fe^2+^-RBF^−^ complex may be ascribed to the low E^0^ value.

**Fig. 3:**
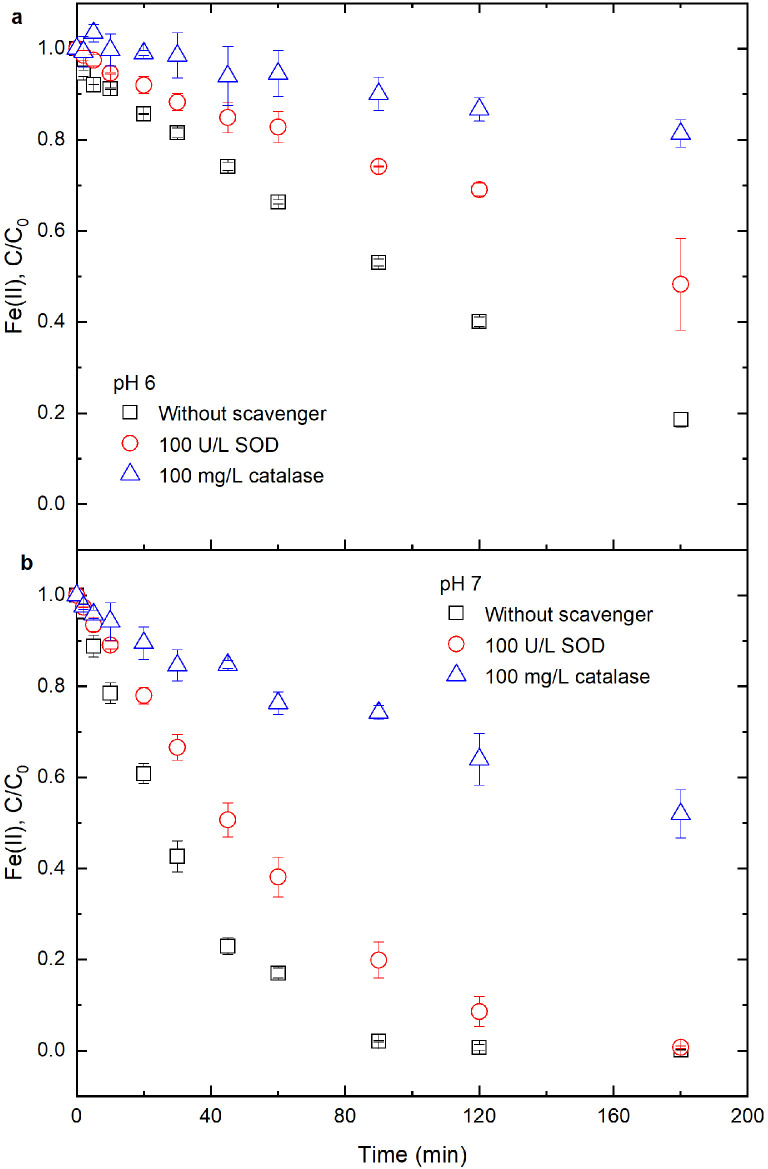
**Effects of SOD and catalase on Fe(II) oxidation in Fe(II)-RBF system at different solution pH.** Initial conditions: SOD, catalase and solution pH specified in panels (a,b), 17.8 μM Fe(II), 10 μM RBF, 10 mM NaCl and 20 mM buffer under oxic conditions.

The quenching experiments show that ·O_2_^−^ and H_2_O_2_ were involved in Fe(II) oxidation in the inorganic Fe(II) and Fe(II)-RBF system (Figs. 3 and S7). However, compared with inorganic Fe(II), less Fe(II) is oxidized by ·O_2_^−^ in the Fe(II)-RBF system. To interpret this discrepancy, we propose that Fe^2+^-RBF^−^ complex oxidation by O_2_ may produce ·RBFOO^−^ (reaction B18 in Table 1) instead of ·O_2_^−^, then ·RBFOO^−^ is further reduced by inorganic Fe(II) and Fe^2+^-RBF^−^ complexes to generate RBFHOOH (reaction B21 in Table 1). RBFHOOH is decomposed to H_2_O_2_ and RBF (reaction B4 in Table 1). Our speculation is partly supported by previous studies that a radical pair (·RBFHOO^−^) is produced during the oxidation of RBFH^−^ by O_2_ [14,21].

**Fig. 4:**
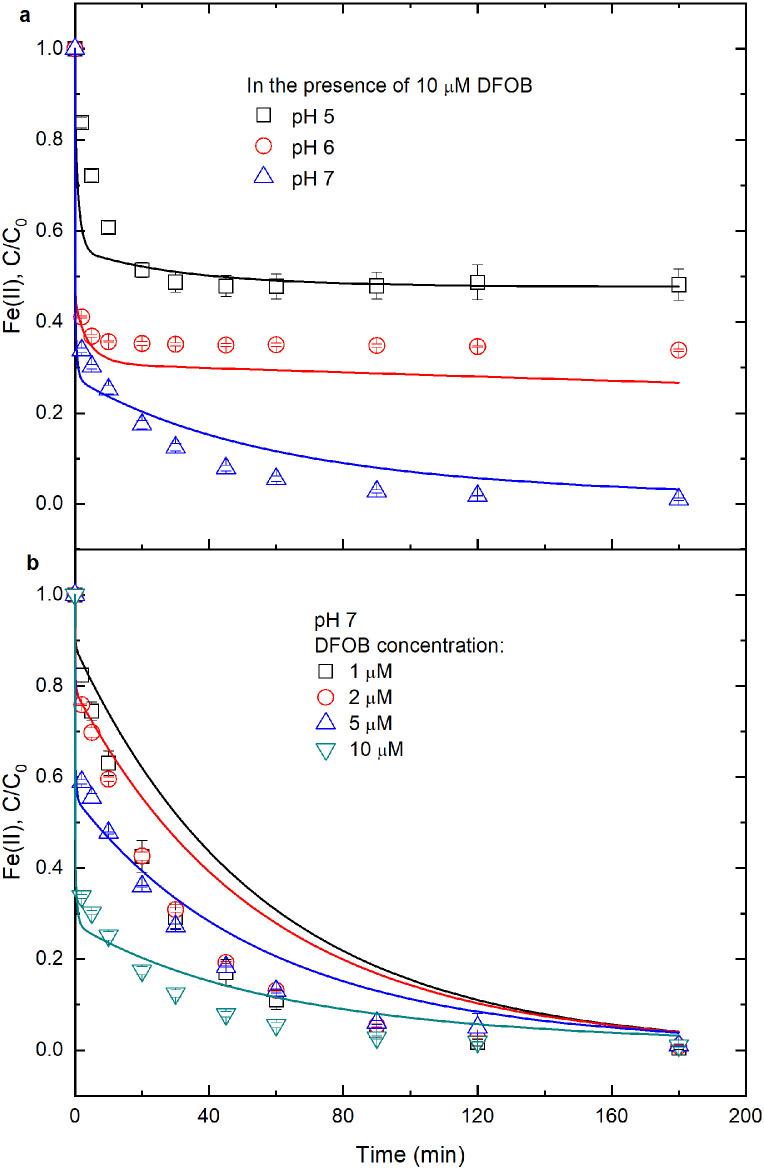
**Effect of DFOB on Fe(II) oxidation by O_2_.** Initial conditions: variable solution pH and DFOB concentration specified in panels (a,b), 17.8 μM Fe(II), 10 mM NaCl and 20 mM buffer under oxic conditions. Points are the average values from duplicate experiments; lines are the modeled curves.

**Fig. 5:**
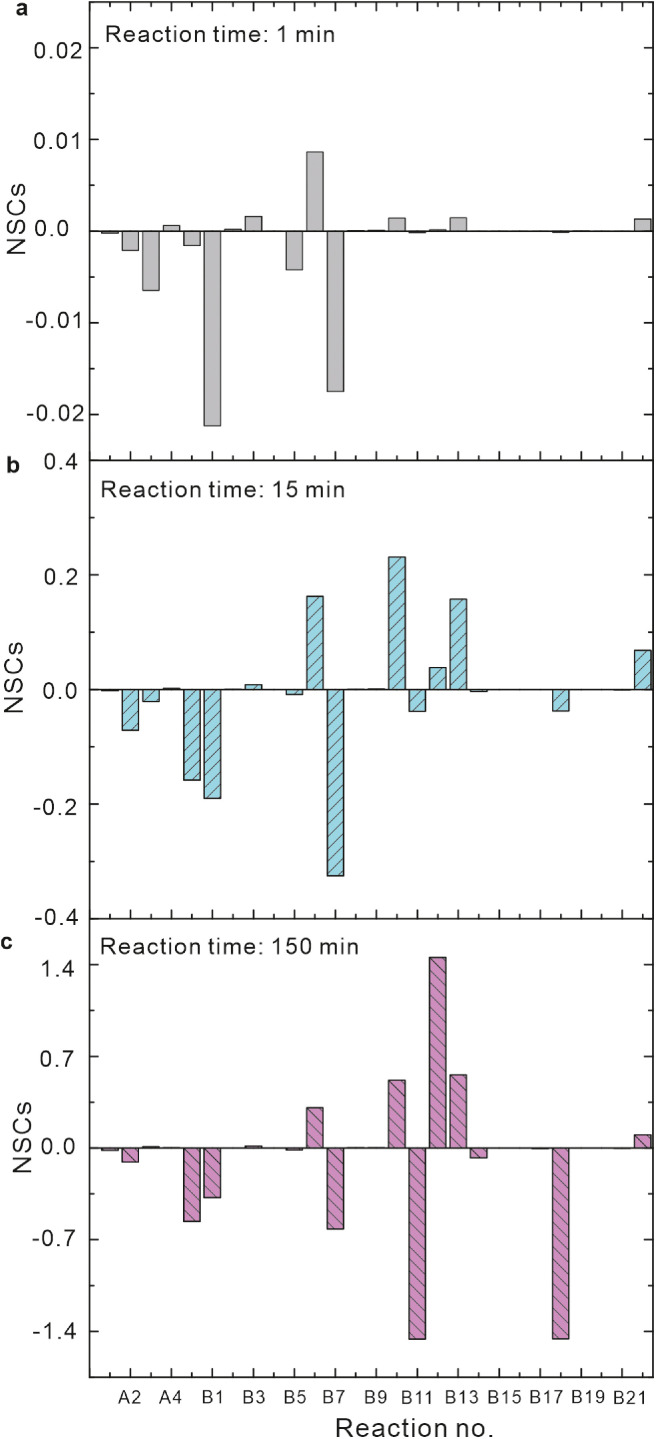
**Normalized sensitivity coefficients (NSCs) at different time for Fe(II) oxidation in Fe(II)-RBFH_2_ system.** Initial conditions: 17.8 μM Fe(II), 10 μM RBFH_2_ and pH 6.

**Fig. 6:**
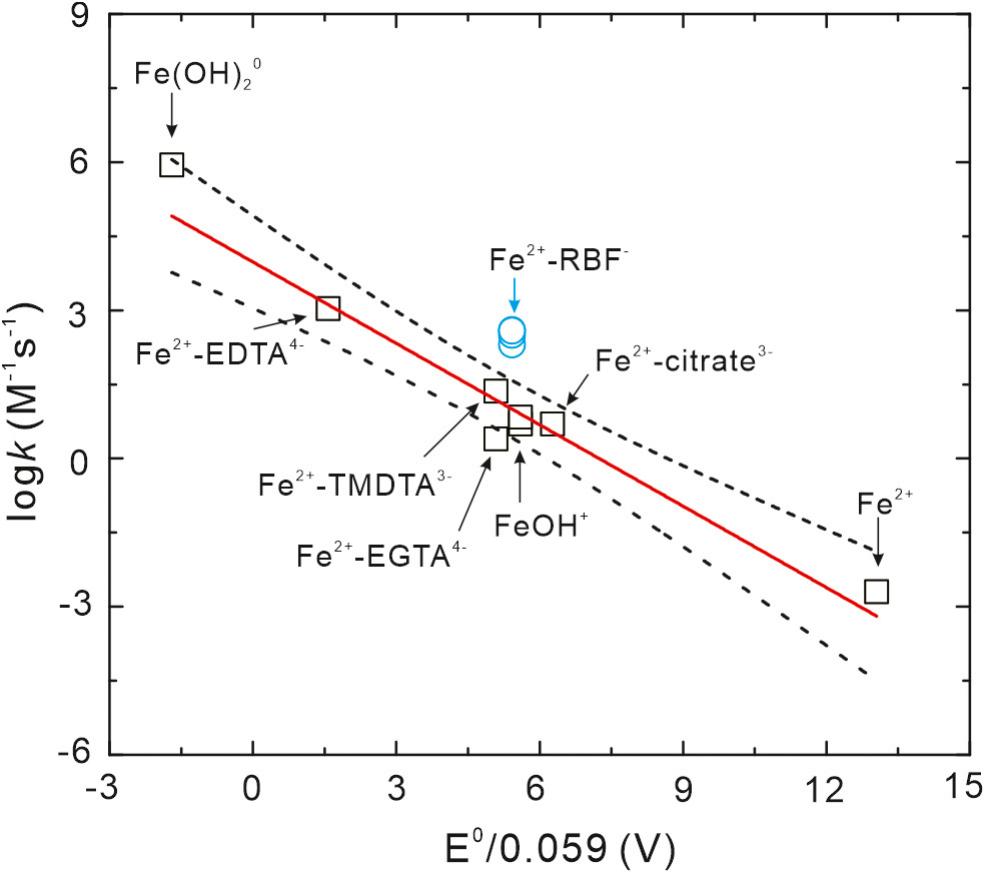
**Correlation between the second-order rate constants for oxidation of Fe(II) by O_2_ and standard reduction potentials (E^0^) for the Fe(III)/Fe(II) redox couples.** Black square represents the previously reported data for inorganic/complexed Fe(II) species [7], [33], [36], [41]. The solid line (slope = −0.54, intercept = 3.98, R^2^ = 0.91, *n* = 8) is the linear fit curve based on the previously reported data and the dotted lines are the upper and low limits for 95% confidence intervals. The terms of EDTA, TMDTA, EGTA were the abbreviation for ethylenediaminetetraacetic acid, trimethylenediamine- N,N,N’,N’-tetraacetic acid and ethylene glycol tetraacetic acid, respectively.

#### 4.2.3 Relative importance of each pathway on Fe(II) oxidation

**Fig. 7:**
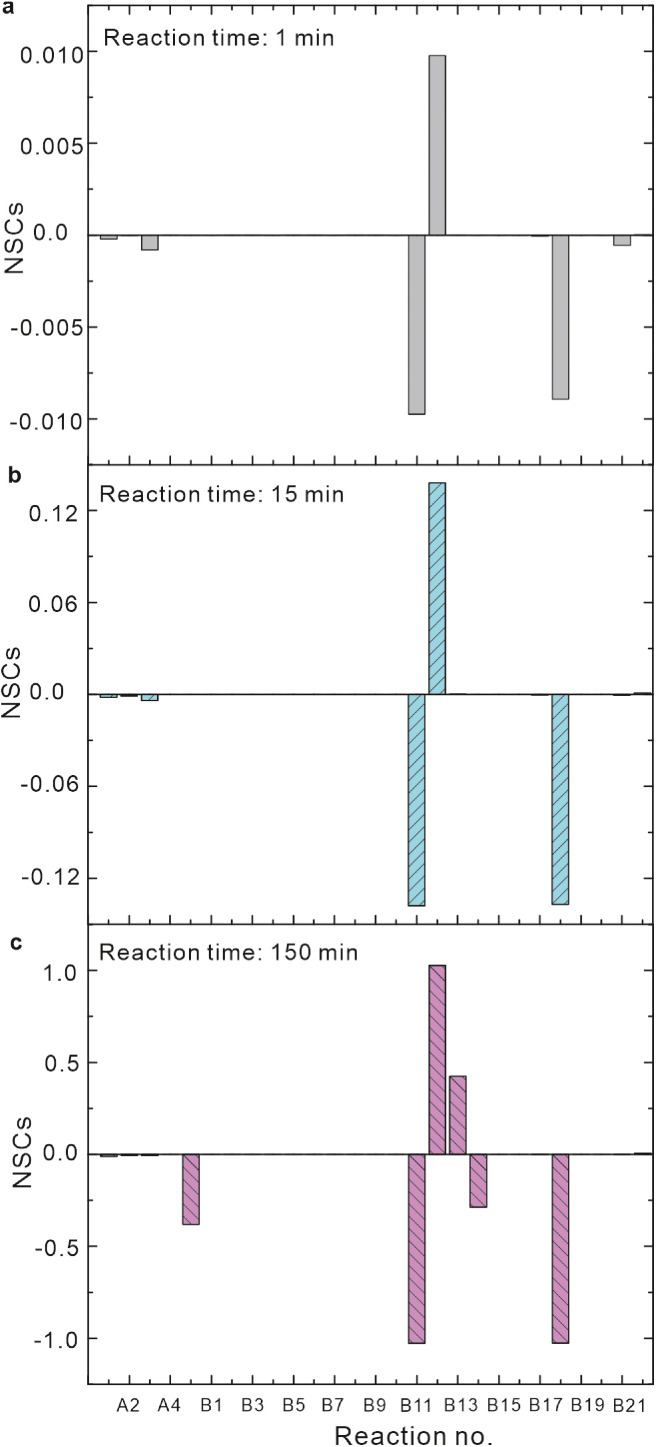
**NSCs of reactions at different time for Fe(II) oxidation in Fe(II)-RBF system.** Initial conditions: 17.8 μM Fe(II), 10 μM RBF and pH 6.

As shown in Fig. 7, the oxidation of inorganic Fe(II) by RBF and ·RBFH (reactions B15‒B16) and the autodecomposition of Fe^2+^-RBF^−^ complex (reaction B17) had negligible influence on Fe(II) oxidation due to the low NSC values for these reactions. By contrast, the formation and oxidation of Fe^2+^-RBF^−^ complex (Reactions B11 and B18) generated the largest positive NSC values (Fig. 7). Hence, the ligand complexation of RBF mainly contributed to accelerating Fe(II) oxidation in Fe(II)-RBF system, while electron shuttling slightly contributed.

### 4.3 Mechanism of accelerated Fe(II) oxidation by DFOB

**Fig. 8:**
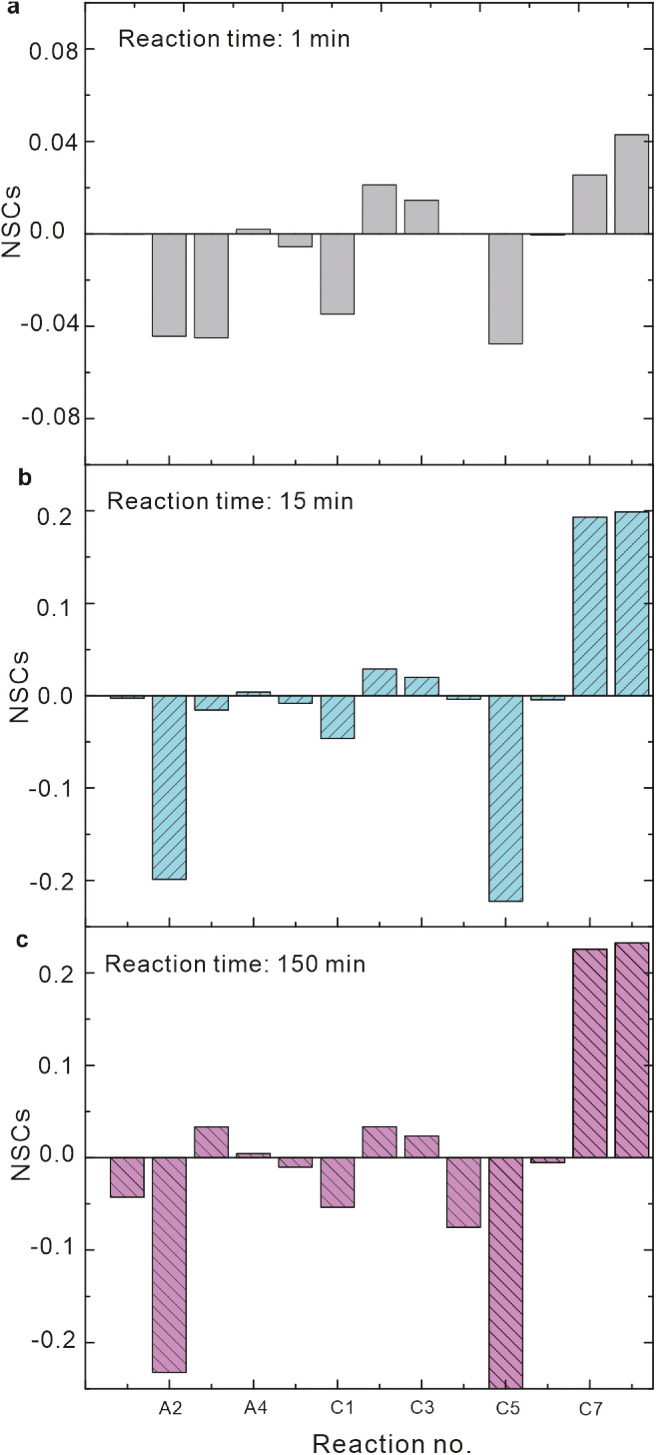
**NSCs of reactions at different time for Fe(II) oxidation in Fe(II)- DFOB system.** Initial conditions: 17.8 μM Fe(II), 10 μM DFOB and pH 6.

Previous studies have shown that DFOB is a strong Fe(II)/Fe(III)-complexing ligand [19,34]. A speciation calculation reflects that Fe^2+^-HDFOB^2−^, Fe^2+^-H_2_DFOB^−^ and Fe^2+^-H_3_DFOB complexes (represents as Fe^2+^-DFOB complex) were produced in the Fe(II)-DFOB system, and the fractions of these Fe^2+^-DFOB complexes increased with increasing both solution pH and DFOB concentrations (Fig. S10). According to the kinetic model, the rate constants for Fe^2+^-DFOB complex oxidation by O_2_ (reaction C5 in Table 1) were derived to be 5 × 10^2^, 3 × 10^4^ and 5 × 10^4^ M^−1^s^−1^ at pH 5, 6 and 7, respectively (Table 1), which were much higher than those for inorganic Fe(II) (reaction A1 in Table 1). Thus, the formation and oxidation of the Fe^2+^-DFOB complex may accelerate Fe(II) oxidation in the Fe(II)-DFOB system. This speculation is supported by the NSC calculation that the reaction C5 generated the largest negative NSC values (Fig. 8).

Based on the LFER approach, the rate constants for Fe^2+^-H_2_DFOB^−^ and Fe^2+^-HDFOB^2−^ complexes oxidation by O_2_ were estimated to be 3.4 × 10^4^ and 2.2 × 10^5^ M^−1^s^−1^, respectively (Section S5), which were close to the model predicted values at pH 6 and 7 but much higher than the model predicted values at pH 5. This discrepancy may be attributed to the following reason. In the kinetic model, all the DFOB complexed Fe(II) species were included, while only two specific Fe(II) species were considered in the LFER.

### 4.4 Mechanism summary and environmental implications

**Fig. 9:**
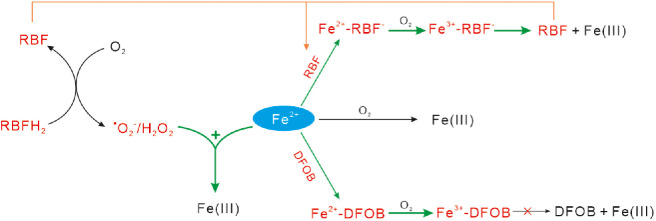
**Summarized mechanisms of RBFH_2_, RBF and DOFB mediated abiotic Fe(II) oxidation**.

The mechanisms responsible for RBFH_2_, RBF and DFOB accelerated Fe(II) oxidation are summarized in Fig. 9. In the Fe(II)-RBFH_2_ system, RBFH_2_ and RBFH^−^ can activate O_2_ to generate ·O_2_^−^ and H_2_O_2_, which then oxidize rapidly the inorganic Fe(II) at the initial stage. The generated RBF can further react with the Fe(II) forming Fe^2+^-RBF^−^ complex, which contributes to the acceleration of Fe(II) oxidation at the last stage. In Fe(II)-RBF and Fe(II)-DFOB systems, RBF and DFOB cannot reduce O_2_, but can chelate Fe^2+^ forming complexed Fe(II). Due to its high reactivity, the formation and oxidation of Fe^2+^-RBF^−^/Fe^2+^-DFOB complexes accelerate Fe(II) oxidation in Fe(II)-RBF and Fe(II)-DFOB systems. However, compared with Fe^2+^-DFOB, the generated Fe^3+^-RBF^−^ complex is unstable, so the Fe^3+^-RBF^−^ complex will further decompose to Fe(III) oxyhydroxides and to release RBF, then continually accelerate Fe(II) oxidation (Section S6).

Redox cycle of Fe(II)/Fe(III) under redox-dynamic conditions is linked to the biogeochemical cycle of carbon, nitrogen, sulfur and phosphorus [1], and impacts the fate and transport of contaminants [3]. Microbial exudate of flavins and siderophores, which are widespread in natural environments, have been documented to play important roles in Fe(III) reduction by Fe-reducing microorganisms under anoxic conditions [1,16]. Our previous study revealed that Fe(II) can be oxidized by flavins under anoxic conditions, especially for alkaline pH conditions [27]. In comparison, the influence of flavins and siderophores on Fe(II) oxidation under oxic conditions has been largely overlooked. This study highlights the acceleration effect of representative flavins of RBFH_2_ and RBF and of representative siderophores of DFOB on Fe(II) oxidation.

Previous studies have shown that natural organic matter (NOM), low-molecular-weight organic acids (LMWOA) and inorganic ligands (such as bicarbonate and phosphate) played important roles in the abiotic oxidation of inorganic Fe(II) in oxygenated surface waters [7,30,[35], [36], [37]]. Also, the rate constants for most NOM or LMWOA complexed Fe(II) oxidation by O_2_ fall in the typical ranges of 1 to 1 × 10^3^ M^−1^s^−1^ [30,38]. In contrast to these complexed Fe(II), the rate constant of DFOB complexed Fe(II) oxidation by O_2_ (5 × 10^2^–5 × 10^4^ M^−1^s^−1^) is higher. However, the concentrations of flavins and DFOB were only at pM‒nM levels in surface waters [24,39], and thus flavins and DFOB may play a comparatively minor role in Fe(II) oxidation in surface water environments. In some micro-environments like biofilms and the rhizosphere, the concentrations of flavins and DFOB reached up to the μM level [16,17,25,40]. In these micro-environments, flavins and DFOB may play a much more prominent role in Fe(II) oxidation. Simulations with the kinetic model suggest that the accelerating effect of RBF, RBFH_2_ and DFOB on Fe(II) oxidation can be significant even at relatively low DO concentrations (see Section S7). For microbial Fe(III) reduction, the bioavailability of Fe(III) is an important factor [1]. Commonly, the newly generated Fe(III) and the complexed Fe(III) are highly reactive. Hence, the flavins/siderophores-accelerated Fe(II) oxidation may benefit further microbial Fe(III) reduction under redox-dynamic conditions.

## 5 Conclusion

This study investigated the influences of RBFH_2_, RBF and DFOB on inorganic Fe(II) oxidation by O_2_ at pH 5‒7. Experimental results show that Fe(II) oxidation was accelerated by the addition of RBFH_2_, RBF and DFOB. Due to the highly reducing reactivity, RBFH_2_ can reduce O_2_ to H_2_O_2_, which then oxidizes Fe(II) rapidly. In addition, the generated RBF can also slightly contribute to the accelerated Fe(II) oxidation in the Fe(II)-RBFH_2_ system. RBF cannot reduce O_2_, but can function as both electron shuttle and ligand to accelerate Fe(II) oxidation in the Fe(II)-RBF system. The relative importance of electron shuttling effect on Fe(II) oxidation is minor due to the slow oxidation of Fe(II) by RBF at pH 5‒7, while the contribution of the formation and oxidation of Fe^2+^-RBF^−^ complexes is predominant. Upon oxidation of the Fe^2+^-RBF^−^ complex, the resulting Fe^3+^-RBF^−^ complex decomposes, generating Fe(III) oxyhydroxides and releasing RBF, which then becomes available again to accelerates Fe(II) oxidation. DFOB is a strong ligand for Fe^2+^/Fe^3+^, so it mediates Fe(II) oxidation through forming the Fe^2+^-DOFB complex. In contrast with the Fe^2+^-RBF^−^ complex and inorganic Fe(II), the rate constant of Fe^2+^-DFOB complex oxidation by O_2_ is more rapid. Because the generated Fe^3+^-DFOB complex is stable and cannot be decomposed to release DFOB, the acceleration effect will be stopped when Fe^2+^-DFOB complex is completely oxidized to the Fe^3+^-DFOB complex. Although the focus of this study has been on the impact of flavins and siderophores on aqueous Fe(II) oxidation, it is still not clear whether the flavins and DFOB can contribute to other Fe species such as adsorbed Fe(II) and Fe(II)-contained minerals that are widespread in subsurface environments.

## Declaration of Competing Interest

The authors declare that they have no conflicts of interest in this work.

## Appendix Supplementary materials

Image, application 1
