# Supplementary material for: Effects of riboflavin and desferrioxamine B on Fe(II) oxidation by O2
Source: Fundam Res. 2021 Oct 21;2(2):208–17. doi: 10.1016/j.fmre.2021.09.012 (PMC11197622; doi:10.1016/j.fmre.2021.09.012)
Supplement: Supplementary file 1 [file mmc1.docx]

Supporting Information for

Effects of riboflavin and desferrioxamine B on Fe(II) oxidation by O_2_

Peng Zhang ^a^, Philippe Van Cappellen ^b, *^, Kunfu Pi ^b, c^, Songhu Yuan ^a, *^

^a^ State Key Laboratory of Biogeology and Environmental Geology, China University of Geosciences, 68 Jincheng Street, East Lake High-Tech Development Zone, Wuhan, Hubei 430078, China

*^b^ Ecohydrology Research Group, Water Institute and Department of Earth and Environmental Sciences, University of Waterloo, Waterloo, Ontario N2L 3G1, Canada*

^c^ Hubei Key Laboratory of Yangze Catchment Environmental Aquatic Science, School of Environmental Studies, China University of Geosciences, 68 Jincheng Street, East Lake High-Tech Development Zone, Wuhani 430078, China

*E-mail: [pvc@uwaterloo.ca](mailto:pvc@uwaterloo.ca) (P. Van Cappellen); [yuansonghu622@cug.edu.cn](mailto:yuansonghu622@cug.edu.cn) (SH. Yuan);

Section S1 Descriptions of reactions and associated rate constants for kinetic modeling

In Table 1 in the main text, reactions A1–A3 describe the oxidation of inorganic Fe(II) by O_2_, •O_2_^-^ and H_2_O_2_, reaction A4 describes the reduction of aqueous Fe(III) by •O_2_^-^, and reaction A5 describes the hydrolysis of aqueous Fe(III). Reactions B1–B2 describe the dissociation and formation equilibrium of RBFH_2_, reactions B3–B4 describe H_2_O_2_ generation through the oxidation of RBFH^-^ by O_2_, reactions B5–B6 describe the equilibrium reaction between RBF/RBFH_2_ and RBF radicals (•RBFH), reactions B7–B8 describe the oxidation of •RBFH by O_2_ and •O_2_^-^, reaction B9 describes the oxidation of RBFH_2_ or RBFH^-^ by •O_2_^-^, reaction B10 describes the reduction of aqueous Fe(III) and Fe(III) oxyhydroxides (represents as Fe(OH)_3_) by RBFH_2_ or RBFH^-^, reaction B11 describes the complexation of aqueous Fe(II) by RBF, reaction B12 describes the dissociation of Fe^2+^-RBF^-^ complex, reaction B13 describes the complexation of aqueous Fe(III) by RBF, reaction B14 describes the dissociation of Fe^3+^-RBF^-^ complex, reactions B15–B16 describe the oxidation of Fe(II) by RBF and •RBFH, reaction B17 describes the autodecomposition of Fe^2+^-RBF^-^ complex, reactions B18–B20 describe the oxidation of Fe^2+^-RBF^-^ complex by O_2_, •O_2_^-^ and H_2_O_2_, reaction B21 describes the oxidation of Fe^2+^-RBF^-^ complex and inorganic Fe(II) by •RBFOO^-^, and reaction B22 describes the reduction of Fe^3+^-RBF^-^ complex by •O_2_^-^. Reactions C1–C4 describe the complexation of Fe^2+^ and Fe^3+^ by DFOB, reactions C5–C7 describe the oxidation of Fe^2+^-DFOB complex by O_2_, •O_2_^-^ and H_2_O_2_, and reaction C8 describes the reduction of Fe^3+^-DFOB complex by •O_2_^-^.

The rate constant for aqueous Fe(III) hydrolysis to Fe(III) oxyhydroxides (reaction A5 in Table 1) was reported to be 3.2×10^5^ M^-1^s^-1^ at pH 6 [1]. In this study, however, the rate constants for aqueous Fe(III) hydrolysis were adjusted to 3×10^6^ M^-1^s^-1^ at pH 5 and 6. This discrepancy may be ascribed to different experimental conditions. In Pham’s work, the initial Fe(III) concentration was only 0.5 nM, which was five orders of magnitude lower than the used Fe concentration herein (17.8 μM). The higher Fe concentration can result in more rapid hydrolysis and precipitation of aqueous Fe(III). Besides, for the sake of simplification, we made the following assumptions. The rate constants for inorganic Fe(II) oxidation by RBF and •RBFH (reactions B15–B16 in Table 1) as well as the autodecomposition of Fe^2+^-RBF^-^ complex (reaction B17 in Table 1) at pH 5 and 6 were equal to these reactions at pH 7; The rate constants for the oxidation of Fe^2+^-RBF^-^/Fe^2+^-DFOB complexes by •O_2_^-^ (reactions B19 and C6 in Table 1) and for Fe^2+^-RBF^-^ oxidation by H_2_O_2_ (reaction B20 in Table 1) were respectively equal to those for inorganic Fe(II) (reactions A2‒A3 in Table 1); The rate constants for the reduction of Fe^3+^-RBF^-^ and Fe^3+^-DFOB complexes by •O_2_^-^ (reactions B22 and C8 in Table 1) were equal to that for inorganic Fe(II) (reaction A4 in Table 1).

Section S2 Evaluation of reactions not included in the kinetic model

In the kinetic model, the reaction for the catalyzed oxidation of Fe(II) by Fe(III) oxyhydroxides, the oxidation of Fe(II), RBFH_2_, RBF and DFOB by •OH, the complexation of Fe^2+^/Fe^3+^ by RBFH_2_/RBFH^-^, the multistep equilibrium reactions for uncomplexed DFOB, Fe^2+^-DFOB and Fe^3+^-DFOB complexes, the oxidation of Fe(II) by DFOB and the reduction of Fe(III) by DFOB were not included. The specific reasons are addressed as follows.

(1) A previous study reported that the threshold concentration of Fe(III) was 50 μM to form Fe(III) oxyhydroxides for accelerated Fe(II) oxidation [2]. However, the total Fe concentration was only 17.8 μM in this study, which was much lower than the threshold value. So the generated Fe(III) oxyhydroxides may negligibly affect Fe(II) oxidation.

(2) •OH is a strong oxidant, which can react with the redox-sensitive elements and most of organic compounds [3]. However, in this study, the concentrations of organic buffer and Cl^-^ were 20 and 10 mM, respectively, which were much higher than those of Fe(II), RBFH_2_, RBF and DFOB. So, •OH may not be an important oxidant for Fe(II), RBFH_2_, RBF and DFOB.

(3) Our previous study has proven that the RBFH_2_/RBFH^-^ is difficult to chelate with Fe^2+^/Fe^3+^ because the tertiary heterocyclic nitrogen atom is occupied by hydrogen atom [4].

(4) In Fe(II)-DFOB system, all of the dissociation and formation equilibrium of uncomplexed DFOB, Fe^2+^-DFOB and Fe^3+^-DFOB complexes are multistep reactions (Table S1), however most of rate constants for these reactions are lacking in literature. To reduce the parameter uncertainty, the following simplifications were used: the DFOB was used to refer to all the uncomplexed DFOB species, Fe^2+^-DFOB complex refers to all the DFOB complexed Fe(II) and Fe^3+^-DFOB complex refers to all the DFOB complexed Fe(III). The same simplification treatment for the complexation of Fe^3+^ by DFOB was also used in previous studies [1, 5, 6].

(5) Previous studies have reported that Fe(II) can be oxidized to Fe(III) by DFOB at pH >4 [7-9]. To assess the relative importance of this reaction on Fe(II) oxidation, we experimentally measured the rate constant of Fe(II) oxidation by DFOB in a control experiment with the mixture of 17.8 μM Fe(II), 20 μM DFOB, 20 mM buffers and 10 mM NaCl at pH 5 under anoxic conditions. Result shows that the variation of Fe(II) concentration was negligible within 30 minutes (data not shown). However, 9.1 μM Fe(II) was oxidized at pH 5 under oxic conditions (Fig. 4a in the main text). These results suggest that the relative importance of Fe(II) oxidation by DFOB in Fe(II)-DFOB system is minor.

Although Simanova et al. (2010) reported that DFOB may reduce Fe(III) oxyhydroxides at circumneutral pH, the reaction time reached dozens of hours [10]. Thus, the reduction of Fe(III) by DFOB may be negligible within dozens of minutes.

Section S3 Estimation of the apparent rate constants of Fe(II) oxidation

At a fixed pH and oxygen concentration, the inorganic Fe(II) oxidation by O_2_ followed a pseudo first-order kinetic model [12], i.e.,

$\frac{d[\text{Fe}(\text{II})]_{\text{T}}}{dt}=-k_{app}[\text{Fe}(\text{II})]{}_{\text{T}}$ (S1)

where [Fe(II)]_T_ denotes total Fe(II) concentrations (including all Fe(II) species, such as Fe^2+^ and FeOH^+^) and *k_app_* is the apparent rate constant for Fe(II) oxidation. The value of *k_app_* can be derived from the slope of the linear relationship of ln(*C/C*_0_) versus the reaction time (Eq. (S2)).

$\text{ln}(\frac{C}{C_{0}})=-k_{app}t$ (S2)

where *C* and *C*_0_ represent the instantaneous and initial Fe(II) concentrations, respectively, and *t* represents the reaction time.

For the sake of simplification, the pseudo first-order kinetic model was also applied to fit the aqueous Fe(II) concentration versus time curves in Fe(II)-RBFH_2_/RBF systems.

Section S4 Calculation of the quenching efficiency of SOD and catalase on inorganic Fe(II) oxidation.

Figure S7 shows that the additions of SOD and catalase impeded inorganic Fe(II) oxidation. At pH 7, the rate constant of Fe(II) oxidation was 4.0×10^-3^ in the presence of SOD and was 1.7×10^-3^ min^-1^ in the presence of catalase (Fig. S7). Hence, the quenching efficiencies of SOD and catalase on inorganic Fe(II) oxidation were estimated to be 79% (= (1-4.0×10^-3^/1.9×10^-2^)×100%) and 91% (= (1-1.7×10^-3^/1.9×10^-2^)×100%), respectively. At pH 6, the variation of Fe(II) concentration was less than 5% and the rate constants of Fe(II) oxidation in the presence of scavenger could not be obtained from the linear fitting (Fig. S7). So, the quenching efficiencies of SOD and catalase on inorganic Fe(II) oxidation were not calculated at pH 6.

Section S5 Estimation of the rate constants for Fe^2+^-H_2_DFOB^-^ and Fe^2+^-HDFOB^2-^ oxidation by O_2_

According to the linear free energy calculation (Fig. 6 in the main text), the relationship between the rate constant (*k*) for the oxidation of Fe(II) species by O_2_ and its standard reduction potential (E^0^) of Fe(III)/Fe(II) couples can be expressed as,

$\log(k)=-0.54(\frac{E^{0}}{0.059})+3.98$ (S3)

The values of E^0^ for Fe^2+^-H_2_DFOB^-^ and Fe^2+^-HDFOB^2-^ were -0.06 and -0.15 V, respectively (Table S1). Based on Eq. S3, the rate constants for the oxidation of Fe^2+^-H_2_DFOB^-^ and Fe^2+^-HDFOB^2-^ by O_2_ were estimated to be 3.4×10^4^ and 2.2×10^5^ M^-1^s^-1^, respectively.

Section S6 Comparisons of the stability of Fe^3+^-RBF^-^ and Fe^3+^-DFOB and their roles in Fe(II) oxidation

At pH 5‒6, Fe^2+^-RBF^-^ complex predominantly contributes to Fe(II) oxidation in Fe(II)-RBF system, so Fe(II) oxidation will stop when Fe^2+^-RBF^-^ complex is exhausted. As the molar ratio of Fe^2+^ to RBF in Fe^2+^-RBF^-^ complex was 1:1, the maximum amount of Fe(II) oxidation was equal to the initial RBF concentration. However, at pH 6, the slope between the observed decreases in Fe(II) and the initial RBF concentrations was 1.5 (Fig. S11), which was much higher than 1. It is therefore to speculate that RBF may be regenerated. The NSC calculation shows that at time 150 minutes, the aqueous Fe^3+^ hydrolysis and dissociation of Fe^3+^-RBF^-^ complex (reactions A5 and B10) resulted in “Fe(II) oxidation” due to the negative NSC values (Fig. 7 in the main text). However, these reactions cannot consume Fe^2+^, so it may be ascribed to other reasons for the acceleration effect on Fe(II) oxidation.

Previous studies reported that the equilibrium constant for the complexation of aqueous Fe^3+^ by RBF (log*K*) was 3.5 [4], while the value of log*K* for aqueous Fe^3+^ hydrolysis to Fe(OH)_3_ was 15 [13]. Thus, Fe^3+^-RBF^-^ complex was thermodynamically unstable and tended to decompose to Fe(III) oxyhydroxides and RBF. Result of UV-vis absorbance spectra shows that at pH 7, the variation of uncomplexed RBF concentration was negligible during reaction course (Fig. S12). According to the kinetic model, the predicted Fe^3+^-RBF^-^ concentrations increased initially and subsequently decreased, but the concentrations of Fe(III) oxyhydroxides gradually increased with reaction time (Fig. S13). When the uncomplexed RBF is released from Fe^3+^-RBF^-^ complex, it is expected to continually accelerate Fe(II) oxidation.

In comparison with Fe(II)-RBF system, aqueous Fe^3+^ hydrolysis and Fe^3+^-DFOB decomposition played minor influences on Fe(II) oxidation in Fe(II)-DFOB system (Fig. 8 in the main text). The value of log*K* for the complexation of aqueous Fe^3+^ by DFOB was 33.07‒44.15 (Table S1), which was much higher than aqueous Fe^3+^ hydrolysis. Also, the rate constant of Fe^3+^-DFOB complex decomposition was slow. So, Fe^3+^-DFOB was difficult to decompose to Fe(III) oxyhydroxides and to release DFOB. When the uncomplexed DFOB is completely chelated by Fe(III), the acceleration effect on Fe(II) oxidation will diminish. This assumption is supported by experimental observation that the net decreases in Fe(II) concentration at the initial stage was close to DFOB dosage (Fig. 4 in the main text and Fig. S8).

Section S7 Effect of DO concentration on RBF/RBFH_2_/DFOB accelerated Fe(II) oxidation

Previous studies have shown that dissolved O_2_ (DO) concentration is a key factor for Fe(II) oxidation [6, 12]. In subsurface environment, the DO concentration varied greatly [15]. To explore the influence of DO concentration on Fe(II) oxidation, we carried out the calculations for initial DO concentrations of 0.27, 0.1 and 0.05 mM, while keeping the initial Fe(II) concentration at 17.8 μM and the initial RBF, RBFH_2_ and DFOB concentrations at 10 μM. The DO concentration range was in line with that observed in natural systems [15]. As shown in Fig. S14, the presence of RBF/RBFH_2_/DFOB accelerated observably aqueous Fe(II) oxidation at different DO concentrations. For instance, when the initial DO concentration was 0.05 mM, only 44% (7.9 μM) Fe(II) was oxidized within 180 min, while the percentage of Fe(II) oxidation reached by 71% (12.7 μM) and 70% (12.5 μM) with addition of 10 μM RBF and 10 μM DFOB, respectively (Fig. S14). Thus, the presence of riboflavin and DFOB may play an important role in Fe(II) oxidation in subsurface environments.

Table S1 Dissociation constants of DFOB and the stability constants and reduction potentials of Fe(II)/Fe(III)-DFOB complexes.

| *DFOB (L) acid-base chemistry* | log*K* |
| --- | --- |
| H^+^ + L^3-^ ↔ HL^2-^ | 11.45 [11] |
| 2H^+^ + L^3-^ ↔ H_2_L^-^ | 21.44 [11] |
| 3H^+^ + L^3-^ ↔ H_3_L^0^ | 30.62 [11] |
| 4H^+^ + L^3-^ ↔ H_4_L^+^ | 38.94 [11] |
| *Complexation of Fe(II)/Fe(III) by DFOB* |  |
| Fe^2+^ + L^3-^ + H^+^ ↔ Fe^2+^-HL^2-^ | 22.54 [11] |
| Fe^2+^ + L^3-^ + 2H^+^ ↔ Fe^2+^-H_2_L^-^ | 29.41 [11] |
| Fe^2+^ + L^3-^ + 3H^+^ ↔ Fe^2+^-H_3_L^0^ | 34.99 [11] |
| Fe^3+^ + L^3-^ + H^+^ ↔ Fe^3+^-HL^2-^ | 33.07 [11] |
| Fe^3+^ + L^3-^ + 2H^+^ ↔ Fe^3+^-H_2_L^-^ | 43.47 [11] |
| Fe^3+^ + L^3-^ + 3H^+^ ↔ Fe^3+^-H_3_L^0^ | 44.15 [11] |
| *Redox reaction* |  |
| Fe^3+^-HL^2-^ + e^-^ ↔ Fe^2+^-HL^2-^ | E^0^ = -0.15 V^a^ |
| Fe^3+^-H_2_L^-^ + e^-^ ↔ Fe^2+^-H_2_L^-^ | E^0^ = -0.06 V^a^ |

^a^ The data were derived from Eq. S4.

$E^{0}_{Fe(III)-L/Fe(II)-L}=E^{0}_{Fe^{3+}/Fe^{2+}}-0.059\log(K_{Fe(III)-L}/K_{Fe(II)-L})$ (S4)

Table S2 Dissociation constants of RBF and the stability constants of Fe(II)/Fe(III)-RBF complexes.

| *RBF acid-base chemistry* | log*K* |
| --- | --- |
| RBF^-^ + H^+^ ↔ RBF | 6.25 [14] |
| *Complexation of Fe(II)/Fe(III) by RBF* |  |
| Fe^2+^ + RBF^-^ ↔ Fe^2+^-RBF^-^ | 2.15 [4] |
| Fe^3+^ + RBF- ↔ Fe^3+^-RBF^-^ | 9.75 [4] |

Table S3 A summary of the rate constants for Fe(II) oxidation in inorganic Fe(II) and Fe(II)-RBF systems.

| Experimental conditions | pH 5 | | pH 6 | | pH 7 | |
| --- | --- | --- | --- | --- | --- | --- |
|  | *k* (min^-1^) | R^2^ | *k* (min^-1^) | R^2^ | *k* (min^-1^) | R^2^ |
| 17.8 μM Fe(II) + 0 μM RBF | 1.6×10^-5 a^ | - | 3.0×10^-4^ ± 4.6×10^-5^ | 0.79 | 1.9×10^-2^ ± 2.2×10^-4^ | 0.99 |
| 17.8 μM Fe(II) + 1 μM RBF | 3.8×10^-4^ ± 3.5×10^-5^ | 0.91 | 1.6×10^-3^ ± 2.6×10^-5^ | 0.99 | 2.1×10^-2^ ± 6.4×10^-5^ | 0.99 |
| 17.8 μM Fe(II) + 2 μM RBF | 3.6×10^-4^ ± 4.5×10^-5^ | 0.85 | 2.6×10^-3^ ± 6.8×10^-5^ | 0.99 | 2.1×10^-2^ ± 4.1×10^-4^ | 0.99 |
| 17.8 μM Fe(II) + 5 μM RBF | 9.2×10^-4^ ± 4.2×10^-5^ | 0.98 | 4.4×10^-3^ ± 2.0×10^-4^ | 0.98 | 3.2×10^-2^ ± 1.6×10^-3^ | 0.99 |
| 17.8 μM Fe(II) + 10 μM RBF | 9.3×10^-4^ ± 2.9×10^-5^ | 0.99 | 8.4×10^-3^ ± 3.5×10^-4^ | 0.98 | 3.8×10^-2^ ± 1.4×10^-3^ | 0.99 |

^a^ This value was cited from [12].

Table S4 A summary of the rate constants of Fe(II) oxidation in Fe(II)-RBFH_2_ system.

| Experimental conditions | Stage 1 | | Stage 2 | |
| --- | --- | --- | --- | --- |
|  | *k* (min^-1^) | R^2^ | *k* (min^-1^) | R^2^ |
| 17.8 μM Fe(II) +10 μM RBFH_2_, pH 5 | 2.1×10^-2^ ± 1×10^-3^ | 0.98 | 4×10^-3^ ± 1.6×10^-4^ | 0.99 |
| 17.8 μM Fe(II) +10 μM RBFH_2_, pH 6 | 2.1×10^-2^ ± 4×10^-3^ | 0.84 | 6×10^-3^ ± 2.9×10^-4^ | 0.99 |
| 17.8 μM Fe(II) + 10 μM RBFH_2_, pH 7 | 5.2×10^-2^ ± 2×10^-3^ | 0.99 | 2.9×10^-2^ ± 2×10^-3^ | 0.98 |


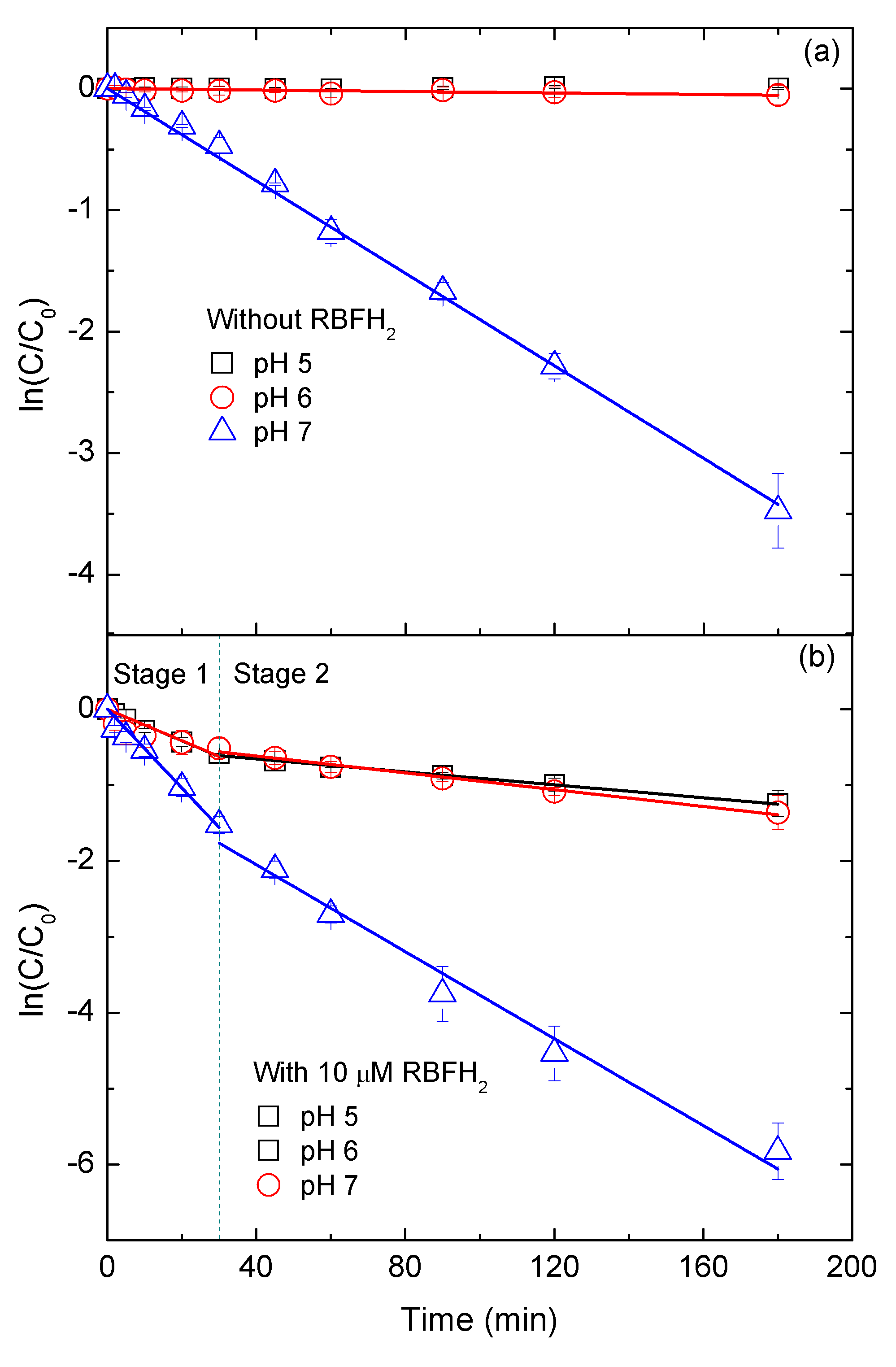


Fig. S1. Plots of ln(*C/C*_0_) versus time for Fe(II) oxidation (a) in the absence of RBFH_2_ and (b) in the presence of RBFH_2_. Initial conditions: RBFH_2_ concentration and solution pH specified in panels (a‒b), 17.8 μM Fe(II), 10 mM NaCl and 20 mM buffer under oxic conditions. The apparent rate constant of Fe(II) oxidation can be derived from the slope (Section S3).


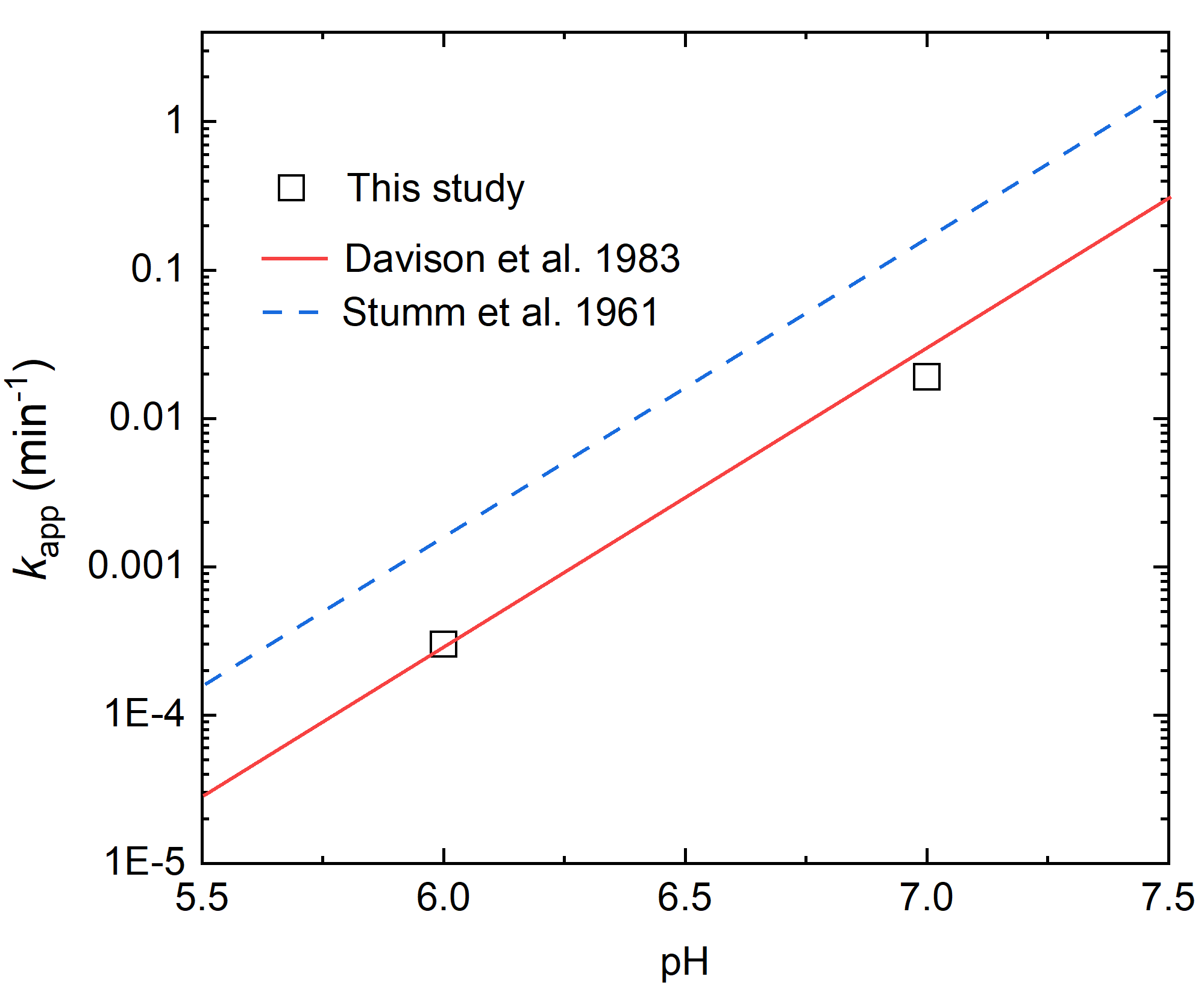


Fig. S2. Comparison of the apparent first-order rate constants of Fe(II) oxidation measured in our study with previously reported rate equations [16, 17].


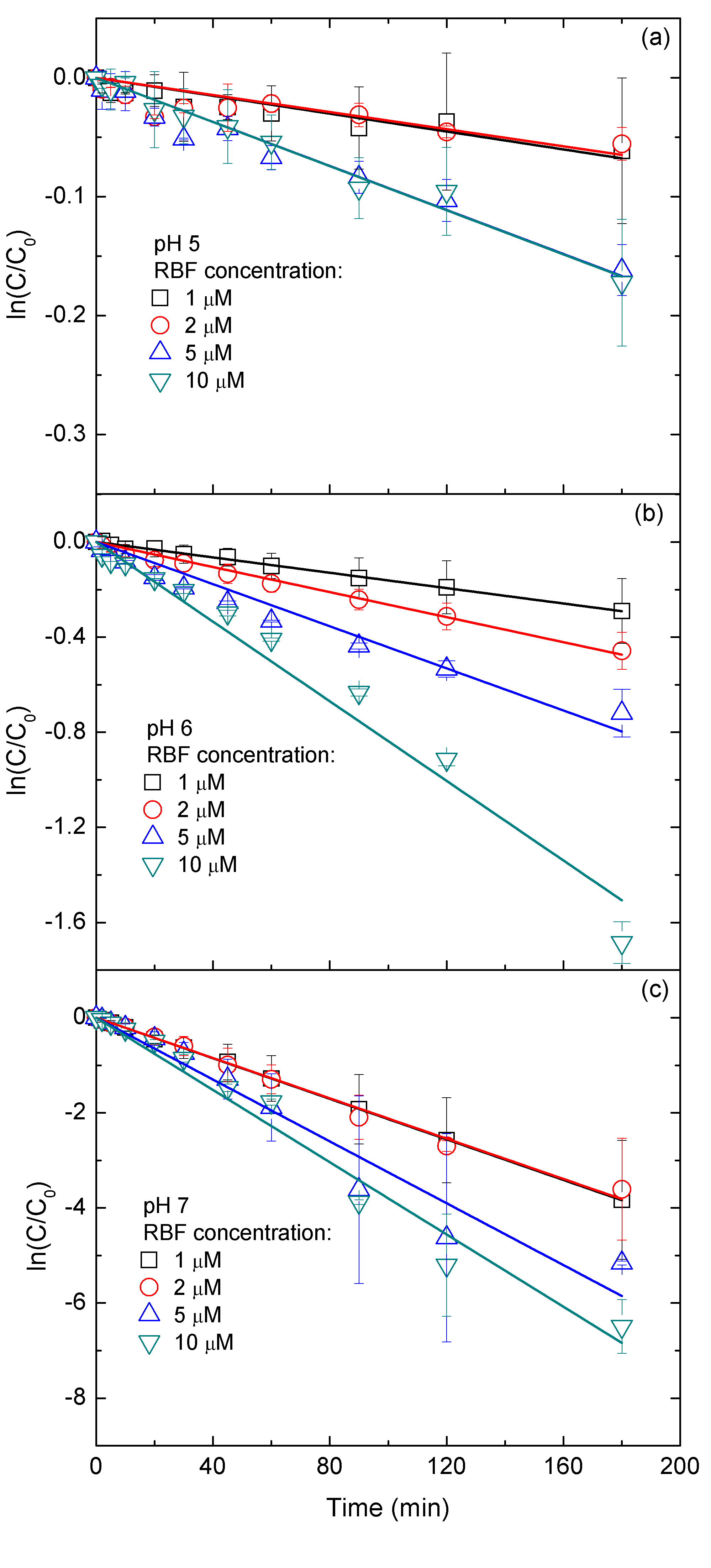


Fig. S3. Plots of ln(*C/C*_0_) versus time for Fe(II) oxidation in Fe(II)-RBF system. Initial conditions: RBFH_2_ concentration and solution pH specified in panels (a‒c), 17.8 μM Fe(II), 10 mM NaCl and 20 mM buffer under oxic conditions. The apparent rate constant of Fe(II) oxidation can be derived from the slope (Section S3).


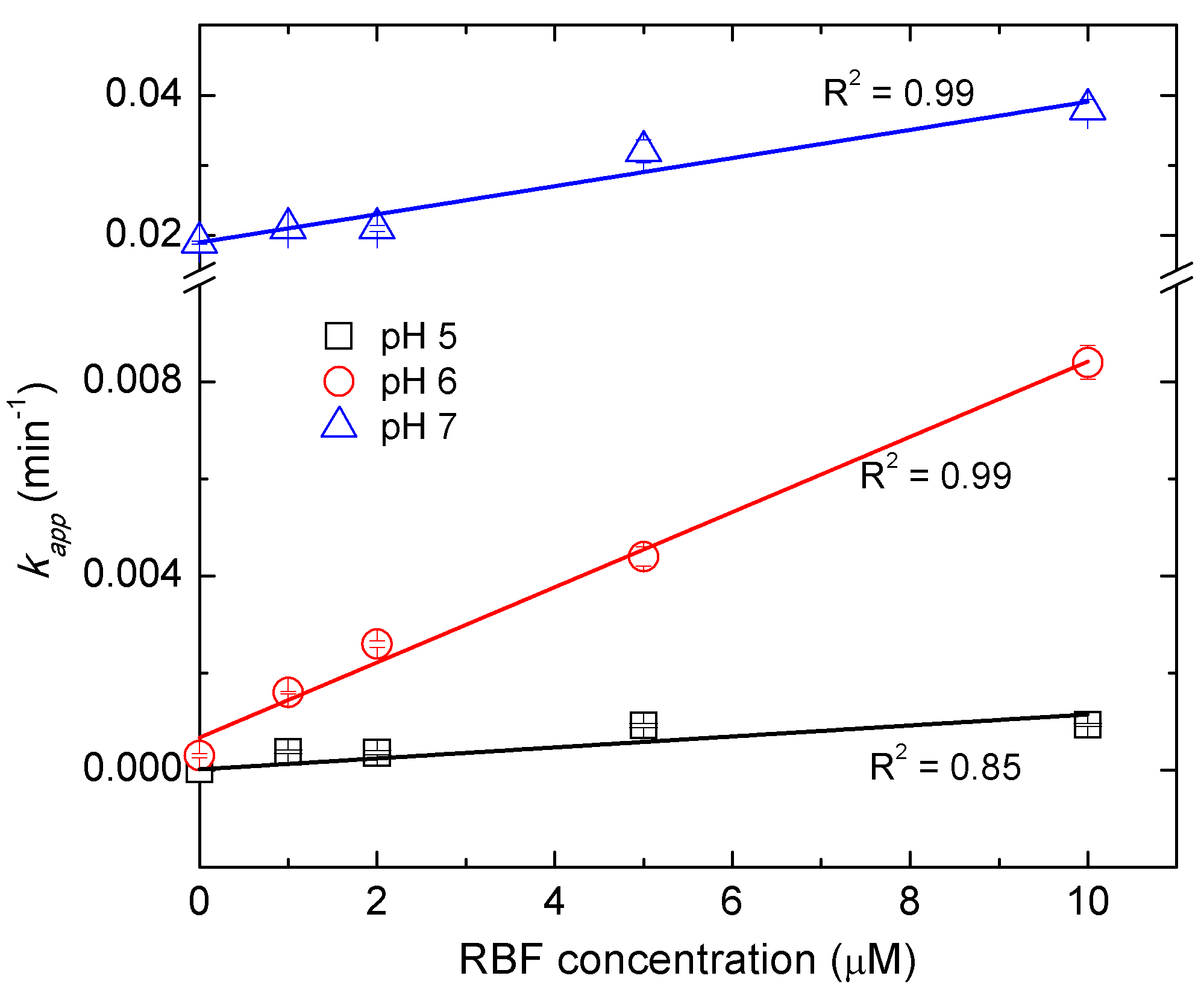


Fig. S4. Dependence of Fe(II) oxidation rate constant (*k_app_*) on RBF concentration. The values of *k_app_* were obtained from Table S3.


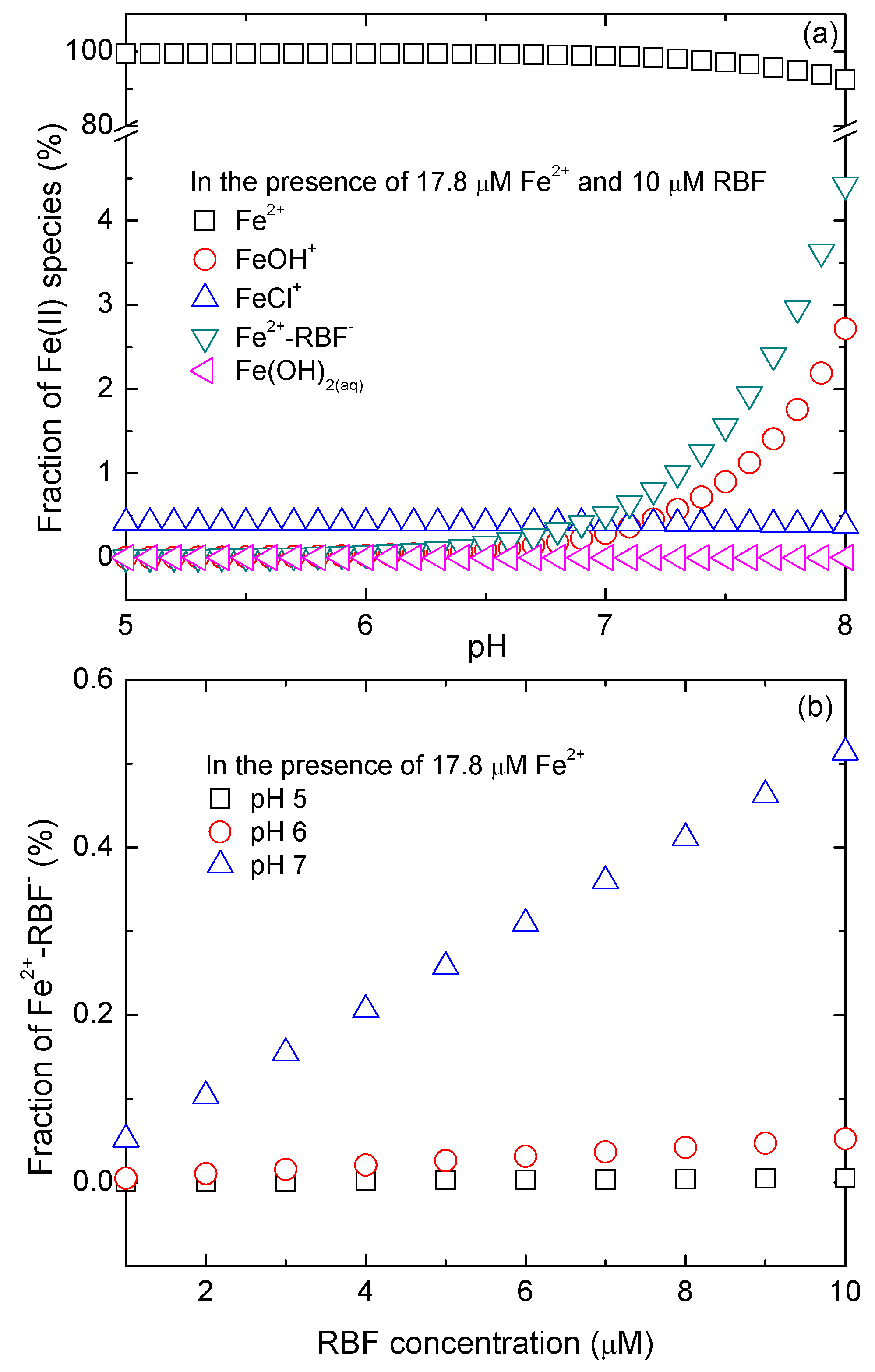


Fig. S5. Fe(II) species distribution as a function of (a) solution pH and (b) RBF concentrations. Initial conditions: variable solution pH and RBF concentration specified in panels (a‒b) and 10 mM NaCl. Calculations were performed by Visual MINTEQ 3.1 [13].


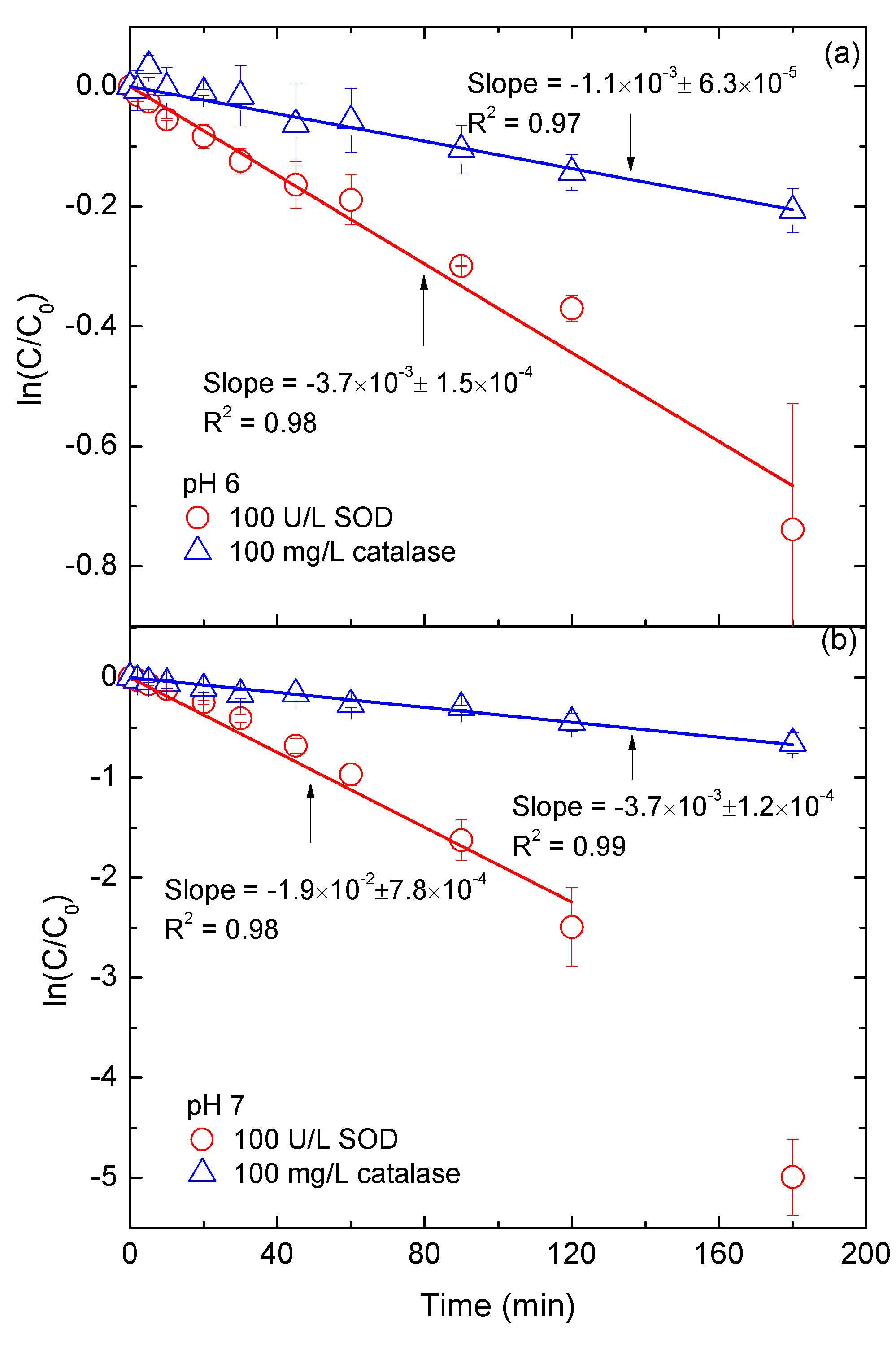


Fig. S6. Plots of ln(*C/C*_0_) versus time for Fe(II) oxidation in Fe(II)-RBF system in the presence of SOD and catalase. The source data were obtained from Fig. 3 in the main text. The apparent rate constant of Fe(II) oxidation can be derived from the slope (Section S3).


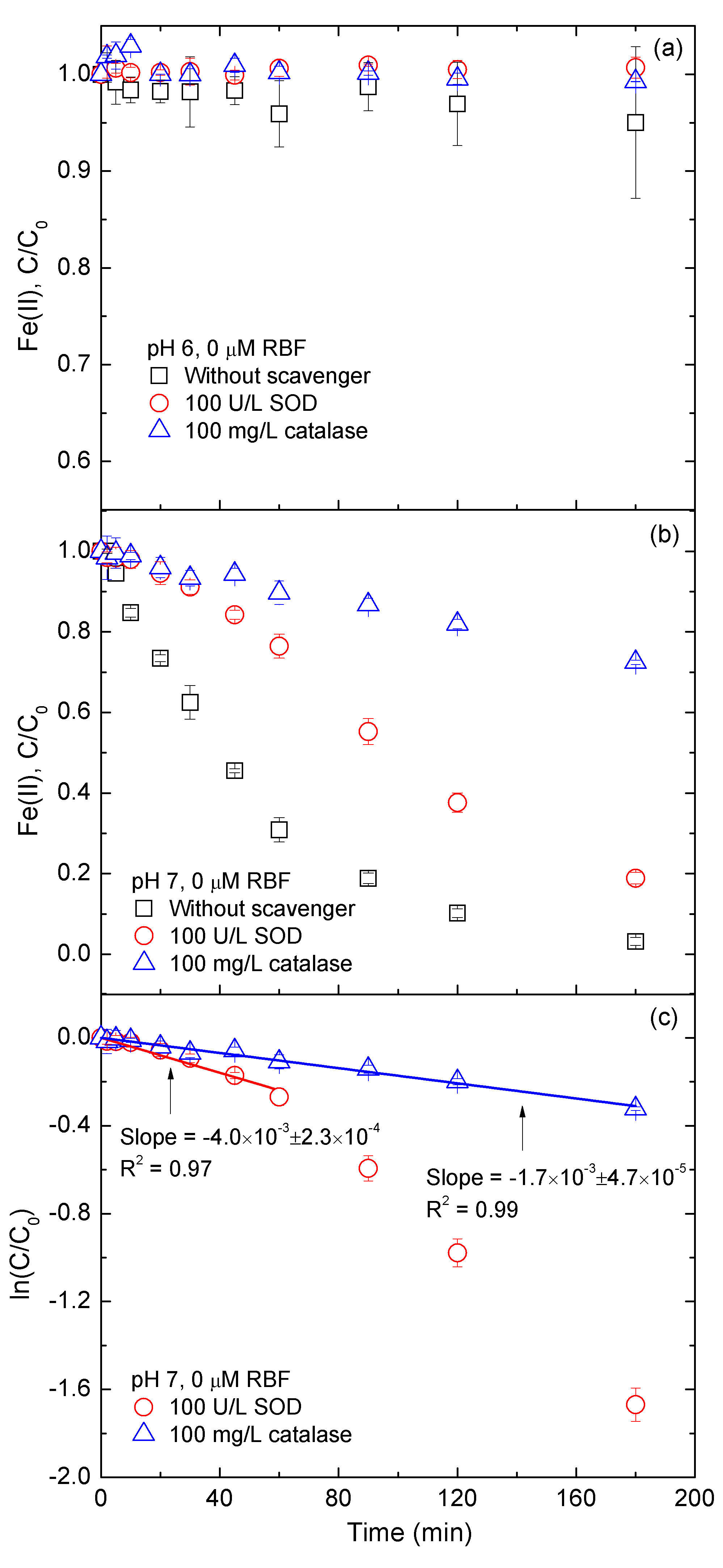


Fig. S7. (a‒b) Effects of SOD and catalase on Fe(II) oxidation in inorganic Fe(II) system and (c) plots of ln(C/C_0_) versus time for Fe(II) oxidation. Initial conditions: SOD, catalase and solution pH specified in panels (a‒c), 17.8 μM Fe(II), 10 mM NaCl and 20 mM buffer under oxic conditions. The apparent rate constant of Fe(II) oxidation can be derived from the slope (Section S3).


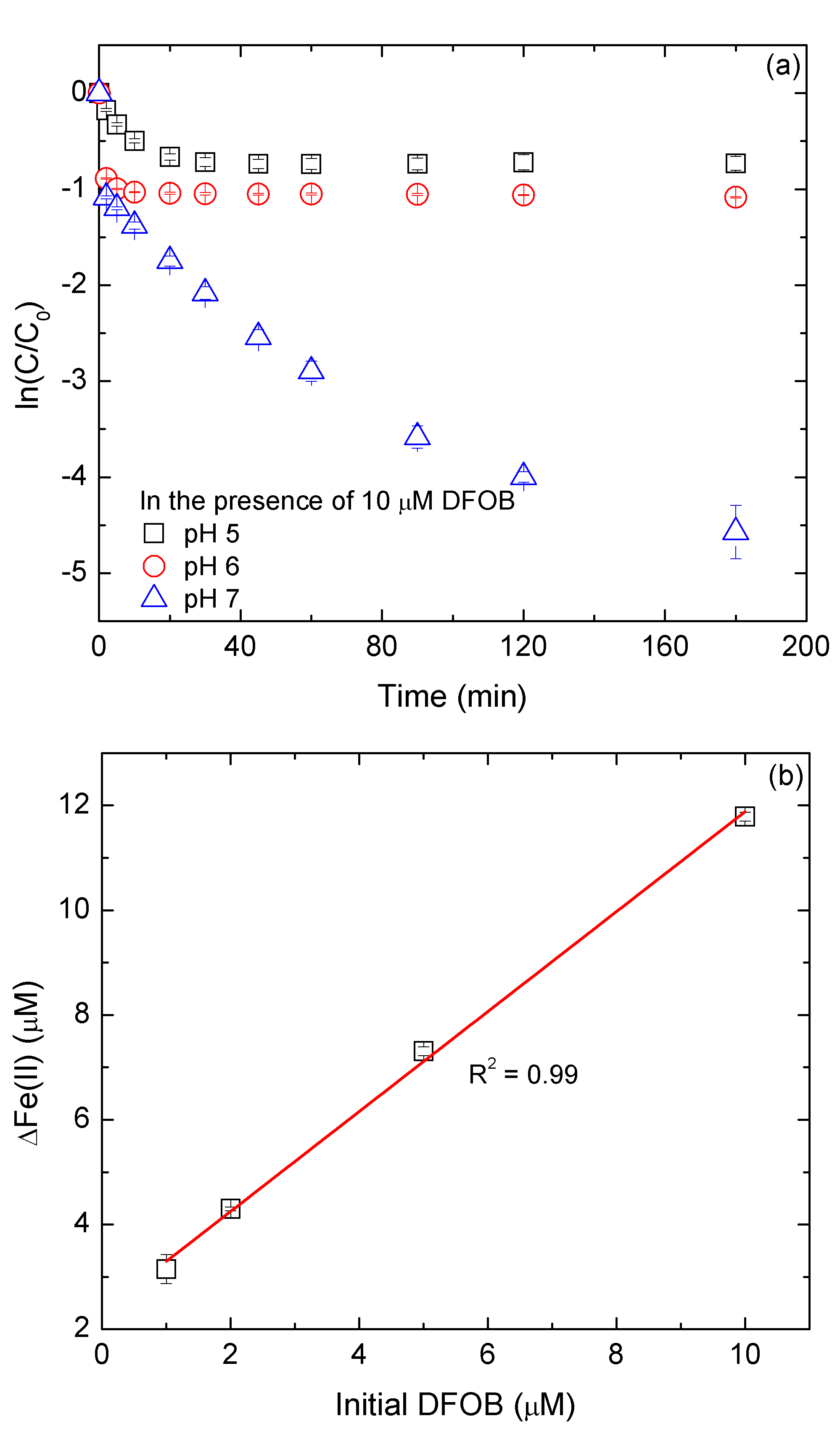


Fig. S8. (a) Plots of ln(*C/C*_0_) versus time for Fe(II) oxidation and (b) relationship between the decreases in aqueous Fe(II) (ΔFe(II)) within initial 2 minutes and initial DFOB dosages. In panel (b) line is best fit linear regression. The source data were obtained from Fig. 4 in the main text.


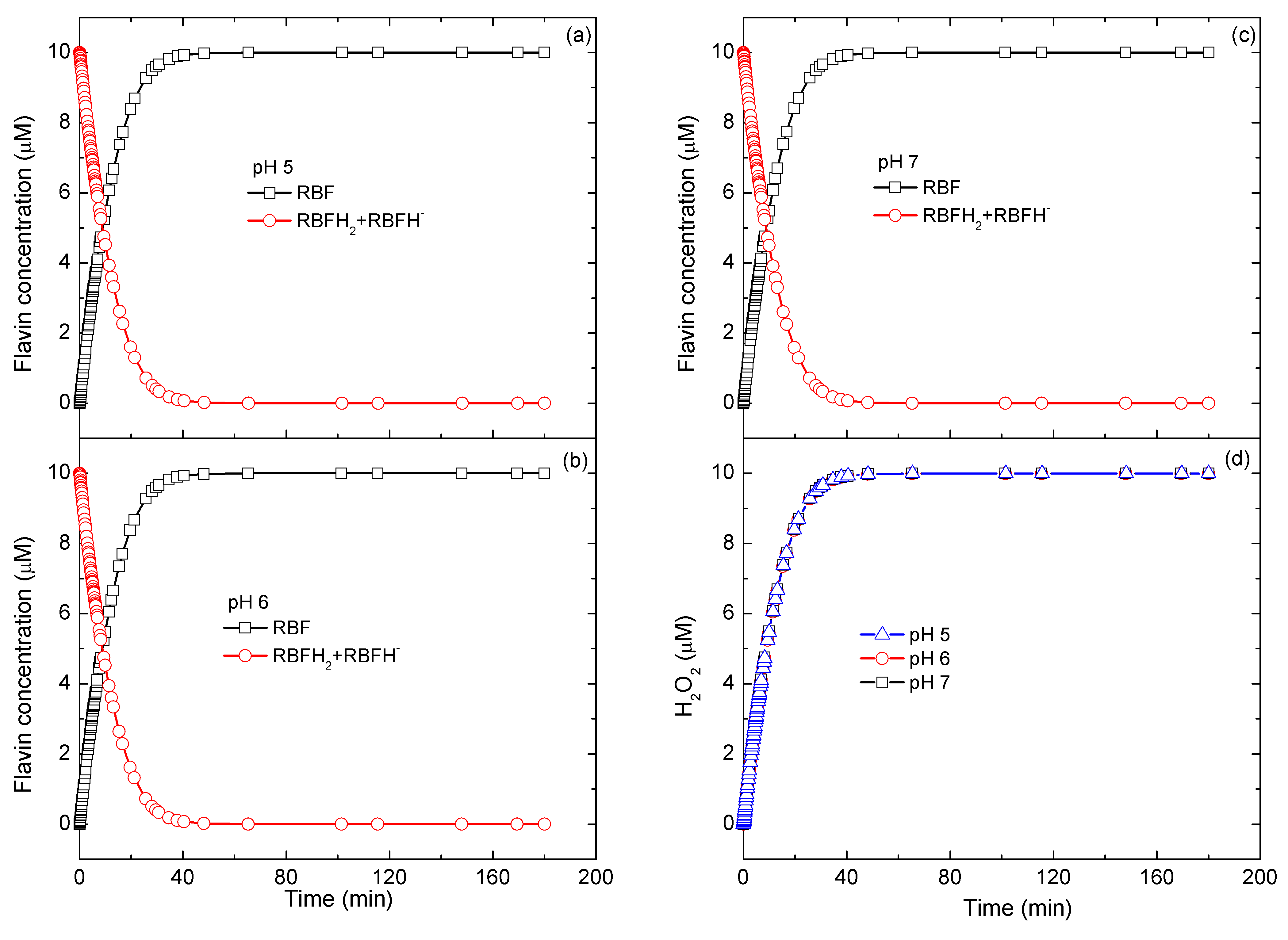


Fig. S9. Modeled concentration trajectories of (a‒c) RBF and RBFH_2_ and (d) H_2_O_2_ as a function of time. Initial conditions: variable solution pH specified in panels, 10 μM RBFH_2_ and 0.27 mM O_2_.


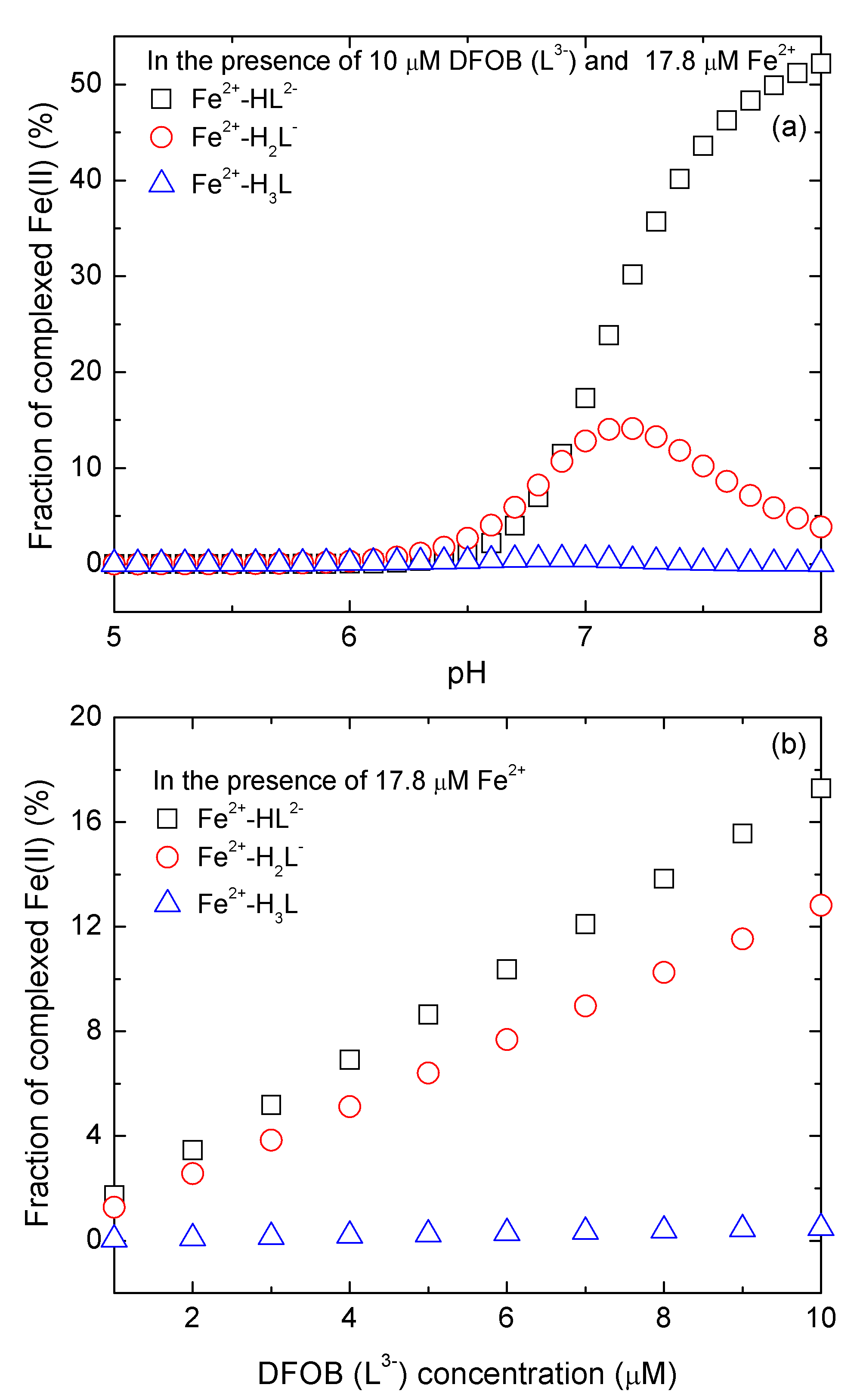


Fig. S10. Variation of complexed Fe(II) to DFOB fractions as a function of (a) solution pH and (b) RBF concentrations. Initial conditions: variable solution pH and DFOB concentration specified in panels (a) and (b) and 10 mM NaCl. Calculations were performed by Visual MINTEQ 3.1 [13].


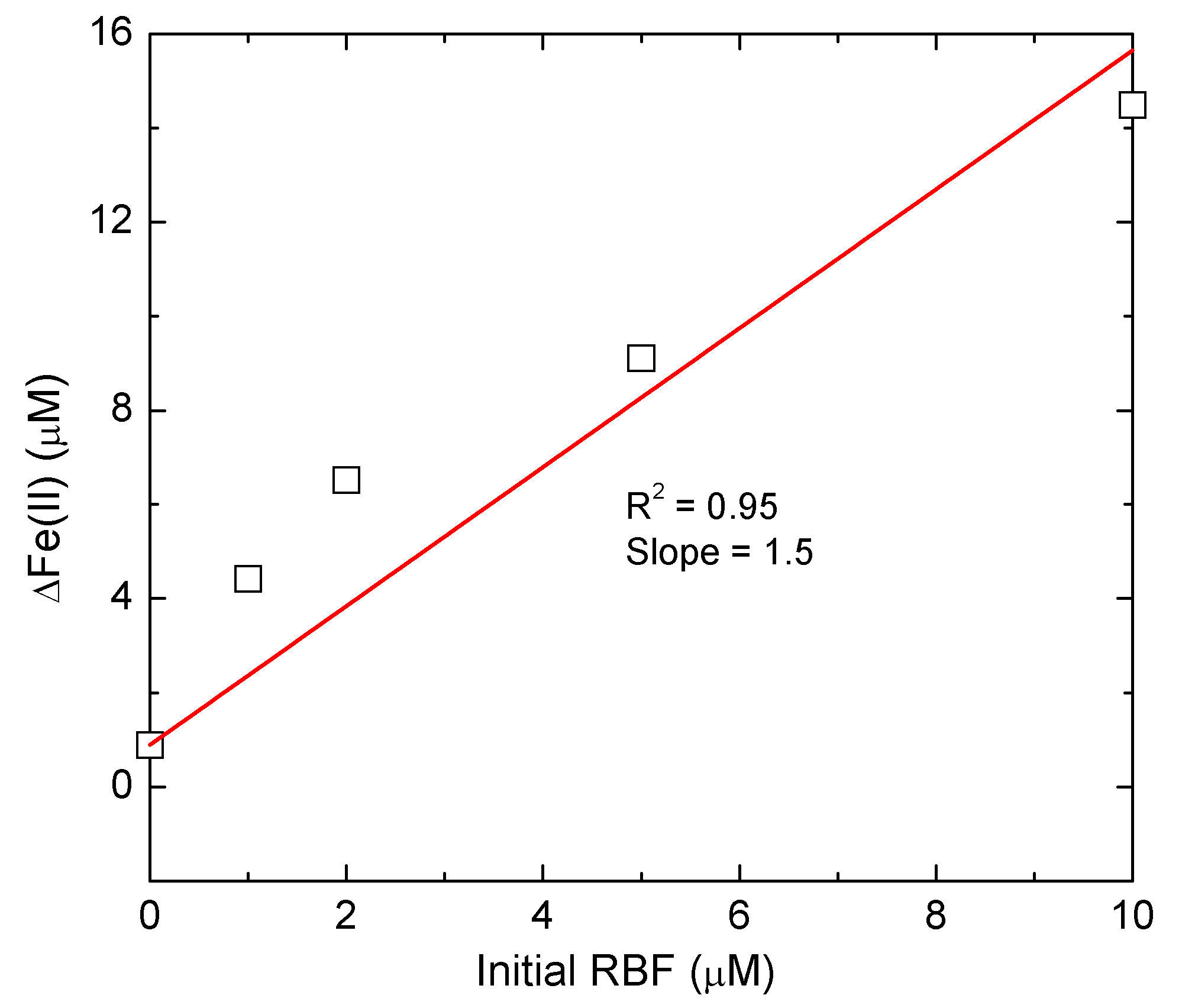


Fig. S11. Relationship between the decreases in aqueous Fe(II) (ΔFe(II)) within initial 180 minutes and initial RBF concentration. Initial conditions: variable RBF concentrations in panel, pH 6, 17.8 μM Fe(II), 10 mM NaCl and 20 mM buffer under oxic conditions. The source data were obtained from Fig. 2 in the main text. The line is best fit linear regression where the value of intercept is set to 0.89.


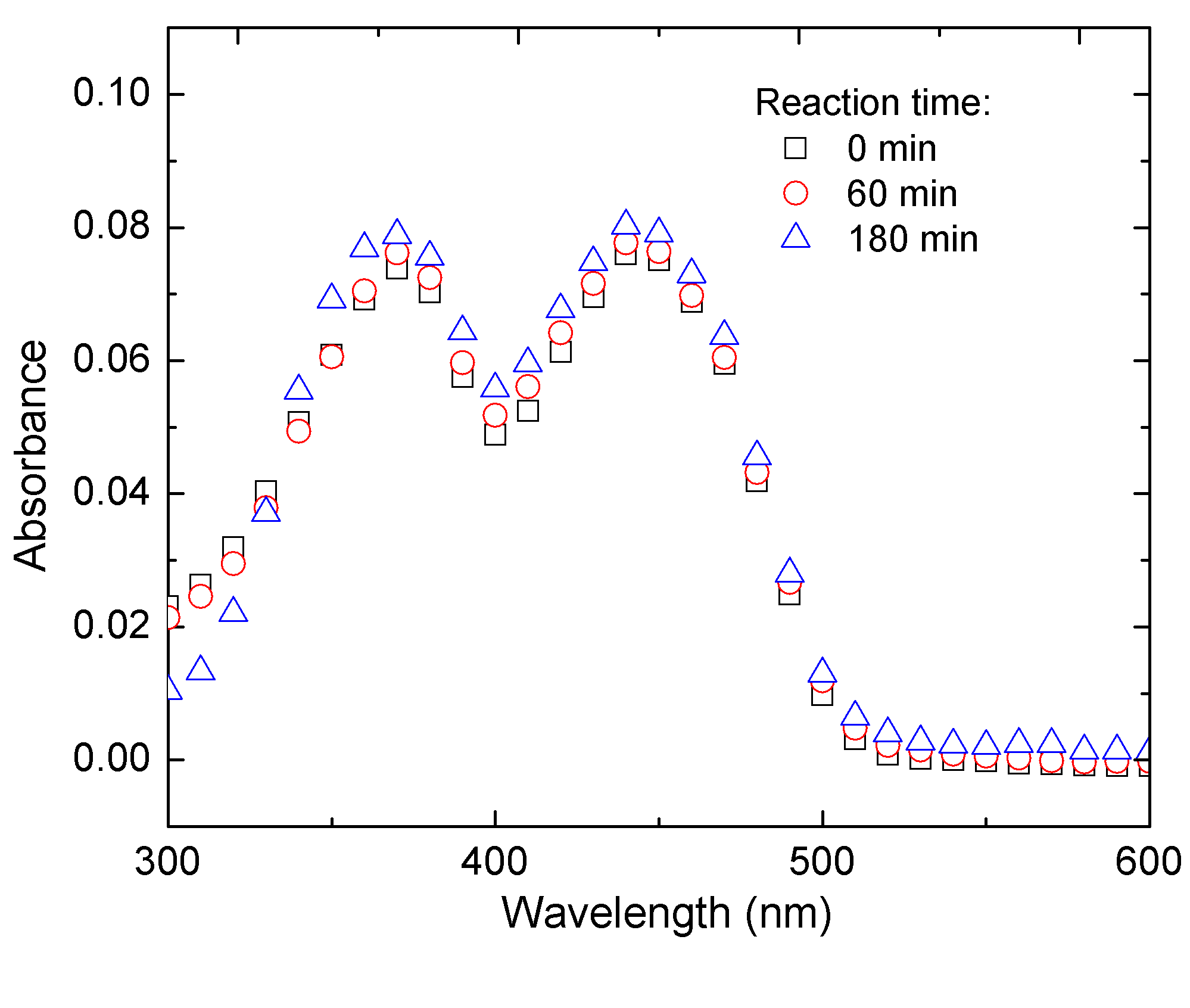


Fig. S12. Variation of UV-vis absorbance spectra for free RBF during reaction course. Initial conditions: 17.8 μM Fe(II), 10 μM RBF and pH 7.


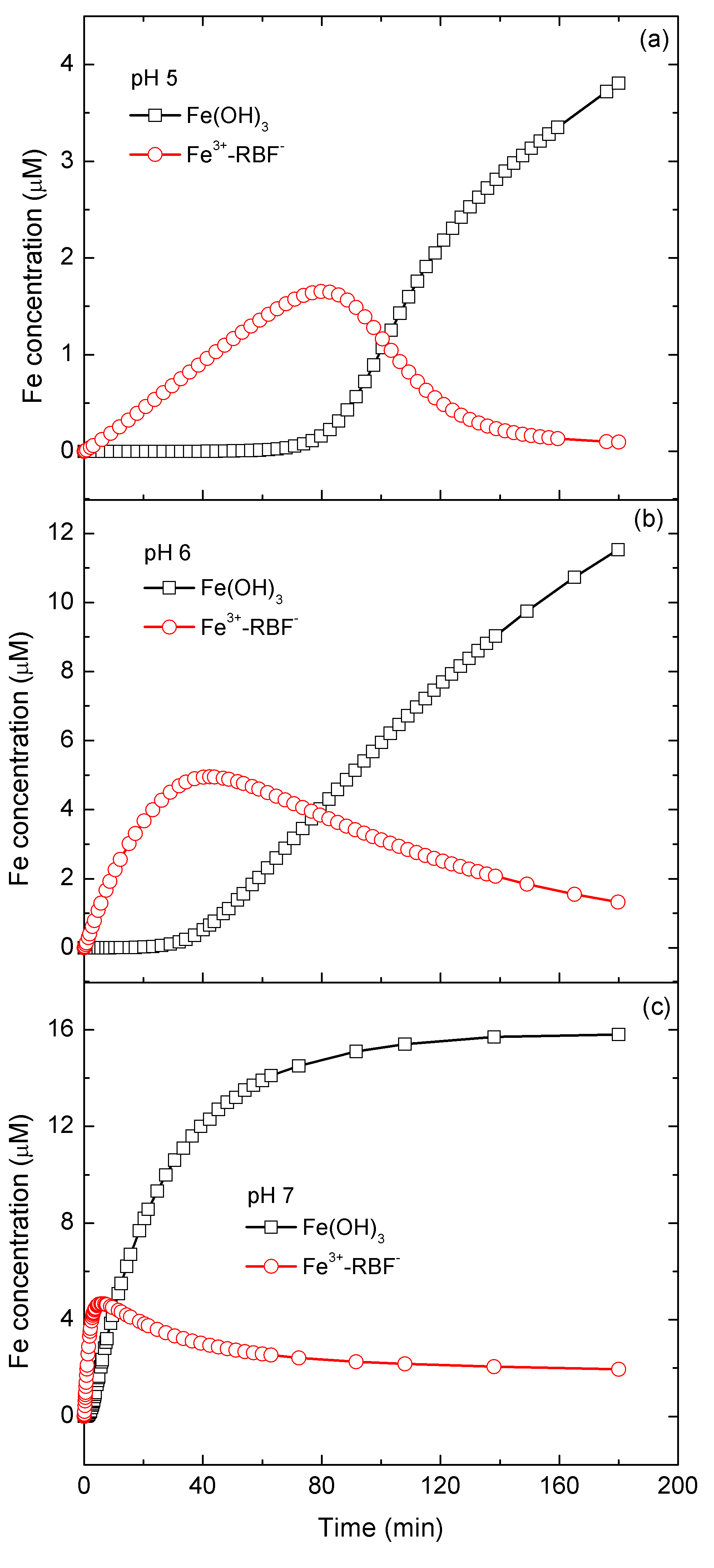


Fig. S13. Modeled concentration trajectories of Fe(OH)_3_ and Fe^3+^-RBF^-^ as a function of time. Initial conditions: variable solution pH specified in panels, 17.8 μM Fe(II), 10 μM RBF and 0.27 mM O_2_.


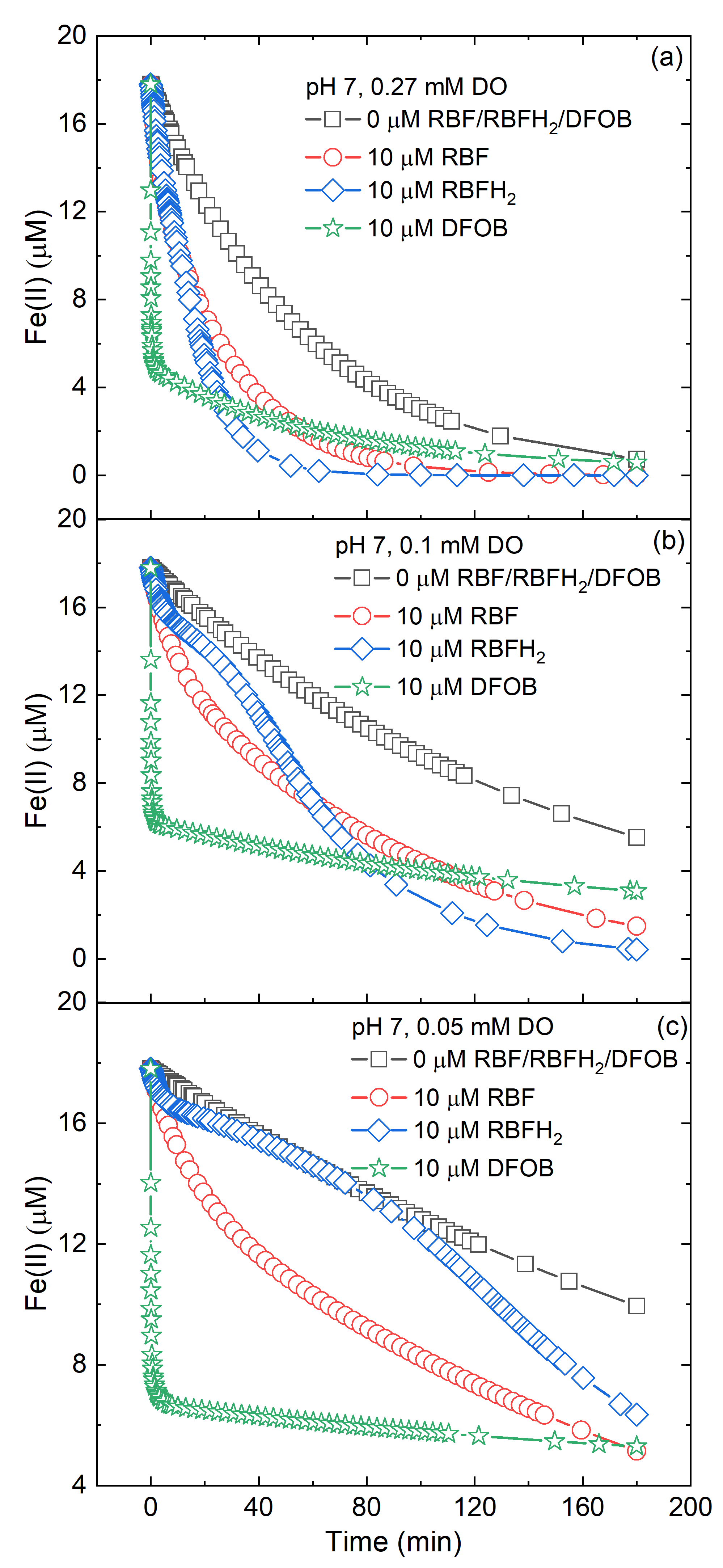


Fig. S14. Modeled trajectory of total aqueous Fe(II) concentration as a function of time. Initial conditions: variable DO concentration specified in panels, 17.8 μM Fe(II), 10 μM RBF/RBFH_2_/DFOB and pH 7.

**REFERENCES**

[1] A. N. Pham, A. L. Rose, A. J. Feitz, T. D. Waite, Kinetics of Fe(III) precipitation in aqueous solutions at pH 6.0–9.5 and 25 °C. Geochim. Cosmochim. Acta 70 (2006), 640–650.

[2] D. W. King, H. A. Lounsbury, F. J. Millero, Rates and mechanism of Fe(II) oxidation at nanomolar total iron concentrations. Environ. Sci. Technol. 29 (1995), 818–824.

[3] G. V. Buxton, C. L. Greenstock, W. P. Helman, A. B. Ross, Critical-review of rate constants for reactions of hydrated electrons, hydrogen-atoms and hydroxyl radicals (^.^OH/^.^O^-^) in aqueous-solution. J. Phys. Chem. Ref. Data 17 (1988), 513–886.

[4] P. Zhang, P. Van Cappellen, K. Pi, S. Yuan, Oxidation of Fe(II) by flavins under anoxic conditions. Environ. Sci. Technol. 54 (2020), 11622–11630.

[5] A. E. Witter, D. A. Hutchins, A. Butler, G. W. Luther, Determination of conditional stability constants and kinetic constants for strong model Fe-binding ligands in seawater. Mar. Chem. 69 (2000), 1–17.

[6] A. L. Rose, T. D. Waite, Kinetics of hydrolysis and precipitation of ferric iron in seawater. Environ. Sci. Technol. 37 (2003), 3897–3903.

[7] E. Farkas, É. A. Enyedy, I. Fábián, New insight into the oxidation of Fe(II) by desferrioxamine B (DFB): spectrophotometric and capillary electrophoresis (CE) study. Inorg. Chem. Commun. 6 (2003), 131–134.

[8] E. Farkas, É. A. Enyedy, L. Zékány, G. Deák, Interaction between iron(II) and hydroxamic acids: oxidation of iron(II) to iron(III) by desferrioxamine B under anaerobic conditions. J. Inorg. Biochem. 83 (2001), 107–114.

[9] D. Kim, O. W. Duckworth, T. J. Strathmann, Hydroxamate siderophore-promoted reactions between iron(II) and nitroaromatic groundwater contaminants. Geochim. Cosmochim. Acta 73 (2009), 1297–1311.

[10] A. A. Simanova, P. Persson, J. S. Loring, Evidence for ligand hydrolysis and Fe(III) reduction in the dissolution of goethite by desferrioxamine-B. Geochim. Cosmochim. Acta 74 (2010), 6706–6720.

[11] D. Kim, O. W. Duckworth, T. J. Strathmann, Reactions of aqueous iron-DFOB (desferrioxamine B) complexes with flavin mononucleotide in the absence of strong iron(II) chelators. Geochim. Cosmochim. Acta 74 (2010), 1513–1529.

[12] D. W. King, Role of carbonate speciation on the oxidation rate of Fe(II) in aquatic systems. Environ. Sci. Technol. 32 (1998), 2997–3003.

[13] J. Gustafsson, Visual MINTEQ, Version 3.1 Division of land and water resources. Royal Institute of Technology, Stockholm, Sweden, (2013).

[14] Z. Shi, J. M. Zachara, L. Shi, Z. Wang, D. A. Moore, D. W. Kennedy, J. K. Fredrickson, Redox reactions of reduced flavin mononucleotide (FMN), riboflavin (RBF), and anthraquinone-2,6-disulfonate (AQDS) with ferrihydrite and lepidocrocite. Environ Sci Technol 46 (2012), 11644–11652.

[15] N. Zhang, X. Bu, Y. Li, Y. Zhang, S. Yuan, Z. Wen, M. Tong, L. Lin, Water table fluctuations regulate hydrogen peroxide production and distribution in unconfined aquifers. Environ. Sci. Technol. 54 (2020), 4942–4951.

[16] W. Davison, G. Seed, The kinetics of the oxidation of ferrous iron in synthetic and natural waters. Geochim. Cosmochim. Acta 47(1983), 67–79.

[17] W. Stumm, G. F. Lee, Oxygenation of ferrous iron. Ind. Eng. Chem. 53 (1961), 143–146.
